# A Hepatitis B Virus-Derived Peptide Exerts an Anticancer Effect via TNF/iNOS-producing Dendritic Cells in Tumor-Bearing Mouse Model

**DOI:** 10.3390/cancers13030407

**Published:** 2021-01-22

**Authors:** Soo-Bin Yang, Mi-Hyun Lee, Bo-Ram Kim, Yu-Min Choi, Bum-Joon Kim

**Affiliations:** 1 Department of Biomedical Sciences, Microbiology and Immunology and Liver Research Institute, College of Medicine, Seoul National University, Seoul 03080, Korea; ysb260@snu.ac.kr (S.-B.Y.); leemh921@snu.ac.kr (M.-H.L.); cym486486@snu.ac.kr (Y.-M.C.); 2 R&D Institute, Cellivery Therapeutics, Inc., K-BIZ DMC Tower, F9, 189 Sungam-Ro, Mapo-Gu, Seoul 03929, Korea; kimbr@cellivery.com

**Keywords:** HBV-derived poly6 peptide, TNF/iNOS-producing DCs (Tip-DCs), type 1 interferon (IFN-I), CD40, cancer immunotherapy

## Abstract

**Simple Summary:**

Recently, it has been reported that a tumor necrosis factor (TNF)/inducible nitric oxide synthase (iNOS)-producing dendritic cell (Tip-DC) may play a pivotal role in the anticancer immune response by activating CD8+ T cells in tumor microenvironments. The development of a new immunotherapeutic agent that can enhance the oncolytic effect of Tip-DC has gained increasing attention in the cancer research field. In this study, we introduce a hepatitis B virus-derived peptide, Poly6, which elicited a strong anticancer immune response via enhanced Tip-DC activity. Our findings suggest that Poly6 could be a novel potential adjuvant/co-treatment partner in anticancer immunotherapy approaches.

**Abstract:**

Recently, we reported a 6-mer hepatitis B virus (HBV)-derived peptide, Poly6, that exerts antiviral effects against human immunodeficiency virus type 1 (HIV-1). Here, we explored the immunotherapeutic potential of Poly6 via its administration into dendritic cells (DCs) in a mouse model. Our data revealed that Poly6 treatment led to enhanced production of tumor necrosis factor alpha (TNF-α) and inducible nitric oxide synthase (iNOS)-producing DCs (Tip-DCs) in a type 1 interferon (IFN-I)-dependent manner via the induction of mitochondrial stress. Poly6 treatment in mice implanted with MC38 cells, a murine colon adenocarcinoma line, led to attenuated tumor formation, primarily due to direct cell death induced by Tip-DC mediated nitric oxide (NO) production and indirect killing by Tip-DC mediated cluster of differentiation 8 (CD8) cytotoxic T lymphocyte (CTL) activation via CD40 activation. Moreover, Poly6 treatment demonstrated an enhanced anticancer effect with one of the checkpoint inhibitors, the anti PD-L1 antibody. In conclusion, our data reveal that Poly6 treatment elicits an antitumor immune response in mice, possibly through NO-mediated oncolytic activity via Tip-DC activation and Tip-DC mediated CTL activation. This suggests that Poly6 represents a potential adjuvant for cancer immunotherapy by enhancing the anticancer effects of immune checkpoint inhibitors.

## 1. Introduction

Since it has fewer side effects than other cancer therapies and can be administered for various cancers, immunotherapy is a promising replacement for other treatments or adjunctive therapies that enhance the treatment efficacy of other cancer therapies [1,2]. However, the ability of tumors to escape the immune system due to the immunosuppressive tumor microenvironment remains a major obstacle in cancer immunotherapy [3]. Local immunosuppression of T cells is a major part of antitumor potential in the tumor microenvironment, and is elicited by cells from the mononuclear phagocytic system, such as myeloid-derived suppressor cells (MDSCs) and tumor-associated macrophages (TAMs) [4,5,6]. Therefore, introducing tools that can reverse the immunosuppressive phenotype is central to harnessing the power of immunotherapeutic strategies.

Dendritic cells (DCs) are potent antigen-presenting cells (APCs) that play a pivotal role in antitumor immune responses [7]. Following the capture of antigens, mature DCs exert strong antitumor effects by inducing a cancer-specific adaptive immune response through the release of Interleukin-12 (IL-12) or cross-presentation of exogenous cancer antigens on major histocompatibility complex class I (MHC-I) molecules [8]. For effective cancer treatment, immunotherapy aims to induce cancer antigen (Ag)-specific immune responses that can induce the death of cancer cells [9]. To achieve this, it is critical to reprogram APCs, particularly DCs, to recover the immune tolerance of cytotoxic T lymphocytes (CTL) and effector T helper (Th) cells specific to cancer Ags in the tumor microenvironment [10,11].

Tumor-associated myeloid cells inhibit antitumor T cell responses in the tumor microenvironment, primarily by inhibitory pathways involving the metabolism of arginine through the regulated expression of two enzymes: arginase 1 (ARG1, encoded by the gene ARG1), which hydrolyzes arginine, and nitric oxide synthase 2 (NOS2, also known as inducible NOS or iNOS), which generates nitric oxide from arginine and oxygen [12]. Even though the precise roles of NOS2 and its reaction products in promoting or controlling cancer are still controversial, a number of findings have been reported, supporting their cancer inhibitory effects, primarily by redirecting APCs to reverse the function of T helper 1 (Th1) responses and CTLs [12] through the release of iNOS-dependent NO production [13].

Recently, Marigo et al. [14] demonstrated that deletion of host iNOS, but not ARG, actually reduced the efficacy of experimental adoptive cell transfer (ACT) in preclinical tumor models. Additionally, they also demonstrated that Tip-DCs (tumor necrosis factor (TNF) and NOS2-producing inflammatory dendritic cells) play a major role in the antitumor activity of ACT in a CD40-CD40L-dependent manner. Tip-DCs mediate NO production via interaction with CTLs, resulting in enhanced tumor-killing activity through TNF and NO production. These findings suggest the potential role of Tip-DC-activating molecules as a method of cancer immunotherapy.

Recently, we introduced a 6-mer hepatitis B virus (HBV)-derived peptide, Poly6, which exerts antiviral effects against HIV-1 by inhibiting p300-mediated acetylation of viral integrase [15]. In this study, we explored the immunotherapeutic potential of Poly6 through its use as a treatment in mouse tumor models, primarily focusing on its capacity to enhance the production of Tip-DCs, and elucidated its underlying antitumor mechanisms.

## 2. Results

### 2.1. Poly6 Treatment Leads to Tip-DC Development from DCs in an IFN-I-Dependent Manner by Evoking Mitochondrial ROS-Mediated Cytosolic Release of Oxidized Mitochondrial DNA

**Figure 1:**
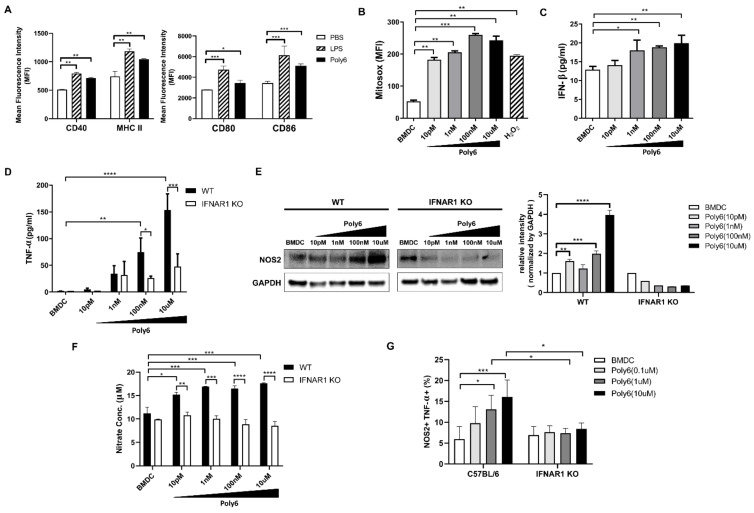
Poly6 treatment leads to tumor necrosis factor (TNF) and nitric oxide synthase 2 (NOS2)-producing inflammatory dendritic cell (Tip-DC) development in DCs in an interferon (IFN)-I-dependent manner by evoking mitochondrial reactive oxygen species (mtROS)-mediated cytosol release of oxidized mtDNA. (**A**) Bone marrow-derived dendritic cells (BMDCs) were starved with reduced –serum minimal essential medium (Opti-MEM) for 30 min and Poly6 (1 µM) was administered for 24 h. Lipopolysaccharide (LPS) (1 mg/mL) was used as positive control. After stimulation with Poly6, maturation of BMDCs was observed by flow cytometry analysis. (**B**) To evaluate mitochondrial ROS, BMDCs were stimulated with Poly6 for 12 h. Additionally, cells were treated with H_2_O_2_ as positive control. Stimulated cells were stained with MitoSOX (5 µM) and assessed by flow cytometry (FACS). (**C**) Supernatants of stimulated BMDCs were collected, and IFN-β ELISA was used to measure type I IFN levels. (**D**) Supernatants of stimulated BMDCs from wild-type (WT) and type I Interferon -alpha/beta receptor deficient (IFNAR1 KO) mice were collected, and TNF-α levels were measured by TNF-α ELISA. (**E**) BMDCs from wild-type C57BL/6 mice and IFNAR1 KO mice were differentiated and exposed to Poly6 stimulation. NOS2 protein was analyzed by Western blotting. (**F**) To detect nitric oxide metabolites, nitrate concentration was assessed by nitrite/nitrate assay kit. (**G**) The development of Poly6-treated BMDCs into Tip-DCs from WT and IFNAR1 KO mice was analyzed by flow cytometry. These results are representative of two independent experiments. Significance differences (* *p* < 0.05, ** *p* < 0.01, *** *p* < 0.001 and **** *p* < 0.0001) among different groups are shown in related figures, and the data are presented as the mean ± standard error of mean (SEM); *n* = 3 biologically independent samples. Student’s *t*-test, one- and two-way ANOVA were used.

Previously, we reported that Poly6, an HBV-derived peptide, induced antiviral effects against HIV-1 by inhibiting the acetylation of HIV-I integrase by downregulating p300 [15]. First, we found that Poly6 inhibits p300 expression in DC2.4 cells (Figures S1A, S11B and S13). This led us to hypothesize that DC modulation by Poly6 may lead to the development of DCs into Tip-DCs in an IFN-I-dependent manner, because higher IFN-β production is known to be a signature of Tip-DCs [16,17]. Given that p300 inhibition can affect the mitochondrial stress response [18,19], we next explored the effect of Poly6 treatment on DC activation or maturation. Our data show that Poly6 treatment induced DC maturation in the DC2.4 cell line and bone marrow-derived dendritic cells (BMDCs), with increased the expression of maturation markers, including C40, CD80, CD86, and MHC II (Figure 1A and Figure S1B). We also found that Poly6 treatment led to mitochondrial reactive oxygen species (mtROS) production (Figure 1B and Figure S1C), resulting in cytosolic release of oxidized mitochondrial DNA (mtDNA) in DC2.4 cells (Figure S1D,E). Oxidized mtDNA can lead to IFN-I production via the cyclic GMP-AMP synthase-stimulator of interferon genes (cGAS–STING) axis [20,21,22], and we checked whether treatment with Poly6 would lead to enhanced expression of both cGAS and STING in DC2.4 cells (Figures S1F, S11B and S14). Our data indicate that Poly6 led to enhanced IFN-I production in DCs in a dose-dependent manner (Figure 1C). Next, we examined the role of increased IFN-I production in Tip-DC development. We found that Poly6 treatment enhanced the production of TNF-α (Figure 1D) and iNOS-dependent NO in BMDCs from wild-type mice but not type I Interferon -alpha/beta receptor deficient (IFNAR1 KO) mice (Figure 1E,F, Figures S11A and S12). Moreover, we found that Poly6 treatment induced the development of BMDCs into Tip-DCs in a dose-dependent manner from wild-type mice but not IFNAR1 KO mice (Figure 1G). We also assessed whether Poly6 treatment can increase the IL-12 production from DCs. Our data showed that Poly6 treatment can lead to IL-12 production from DCs, but in an IFN-I-independent manner (Figure S2A–C). These data suggest that Poly6 leads to Tip-DC development from DC cells in a mitochondrial stress-mediated IFN-I-dependent manner via activation of the cGAS–STING axis.

### 2.2. Poly6 Exerts Anticancer Effects in Mouse Models in an IFN-I Dependent Manner

**Figure 2:**
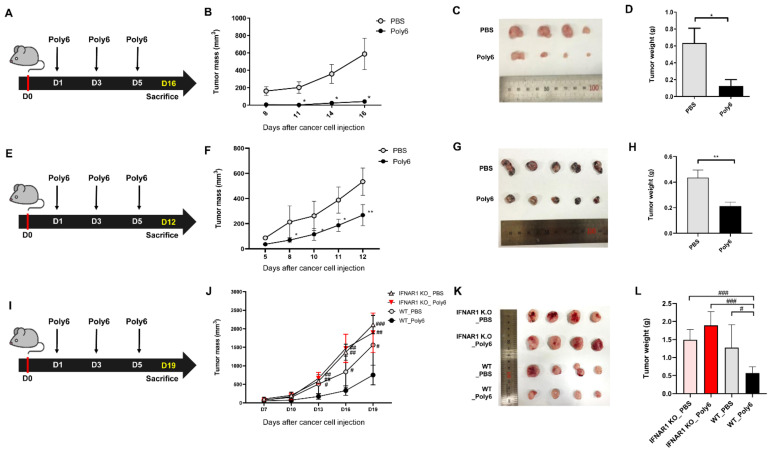
Poly6 exerts anticancer effects in cancer-bearing mouse models. (**A**) Schematic in vivo experimental schedule performed post treatment with Poly6 to verify its therapeutic anticancer effect. MC38 cells (1 × 10^6^) were inoculated by subcutaneous route into C57BL/6 mice (*n* = 4). (**B**) Observing attenuated tumor progression by assessing MC38 tumor growth. (**C**) Images of tumors extracted from MC38 tumor-bearing mice on day 16. (**D**) MC38 colon tumor weight was calculated. (**E**) In vivo experimental schedule of melanoma cancer. B16F10 cells (1 × 10^6^) were subcutaneously injected into C57BL/6 mice (*n* = 5). (**F**) Tumor growth in melanoma tumor. (**G**) Image of B16F10 tumor on day 12. (**H**) Weight of B16F10 tumor tissues was calculated. (**I**) In vivo schematic schedule of MC38 injected interferon knockout mouse experimental model (*n* = 4). (**J**) Tumor growth inhibition by Poly6 in WT C57BL/6 mice but not IFNAR1 KO mice. (**K**,**L**) Tumor tissue image and weight of WT and IFNAR1 KO mice. Tumor mass was calculated using the following formula: width × width × length × 0.52, and mice with over 1000 mm^3^ of tumor mass were sacrificed by CO_2_ asphyxiation. These results are representative of two independent experiments. Significance differences (* *p* < 0.05 and ** *p* < 0.01) among different groups are shown in related figures, and the other significance differences (# *p* < 0.05; ## *p* < 0.01 and ### *p* < 0.001) are used to compare with the Poly6-treated wild-type C57BL/6 mouse group. The data are presented as the mean ± standard error of mean (SEM) of the mice. Student’s *t*-test, one- and two-way ANOVA were used.

Next, we investigated whether Poly6, which could induce Tip-DC and DC maturation in an IFN-I-dependent manner, also had potential as an anticancer vaccine. First, we checked whether Poly6 can exert a direct anti-cancer effect on cancer cells. We could not find any direct anticancer potential of Poly6 on various cancer cells, suggesting that the anti-cancer effect of Poly6 may be dependent on an indirect mechanism (Figure S3A–D). We evaluated the anticancer potential of Poly6 in C57BL/6 mice injected with MC38 mouse colon cancer [23], B16F10 mouse melanoma cancer, or PanO2 mouse pancreatic cancer. To evaluate the therapeutic effects of Poly6, MC38 (1 × 10^6^/100 μL) cells were subcutaneously injected into the right side of each mouse on day 0 and Poly6 (10 µg) was subcutaneously introduced three times on days 1, 3, and 5 after cancer injection (Figure 2A). Our data show that Poly6 significantly inhibited cancer growth and the weight of tumor tissues compared to the phosphate-buffered saline (PBS) -treated group (Figure 2B–D). Similar anticancer effects of Poly6 were also observed in a mouse model implanted with B16F10 mouse melanoma cancer cells (Figure 2E–H) or PanO2 mouse pancreatic cancer cells (Figure S4A–D). Furthermore, we examined the anticancer effect of Poly6 in another MC38-bearing mouse model. In this model, prior to cancer cell seeding (1 day prior to cancer injection), Poly6 was challenged via a subcutaneous route for pre-activation of DCs and further challenged three times at longer time intervals: on days 7, 14, and 24 after cancer injection (Figure S4E). In this model, Poly6-treated mice also exhibited reduced tumor size and weight, as shown in the therapeutic effect model (Figure S4F–H). In addition, tumor incidence was also markedly reduced and survival was significantly increased in Poly6-challenged mice compared to the PBS group (Figure S4I,J). Next, we checked the IFN-I dependency of the anticancer effect of Poly6 by comparing the anticancer effects of treatment between wild-type and IFNAR1 KO mice (Figure 2I). We found that the reduction in tumor mass and weight by Poly6 observed in WT mice was not found in IFNAR1 KO mice (Figure 2J–L). These results suggest that Poly6 has therapeutic IFN- I dependent anticancer potential in cancer mouse models.

### 2.3. Poly6 Exerts Anticancer Effects via Induction of Apoptotic Tumor Cell Death in the Tumor Microenvironment Primarily by Activating CD8 T Cell-Mediated CTL Response

**Figure 3:**
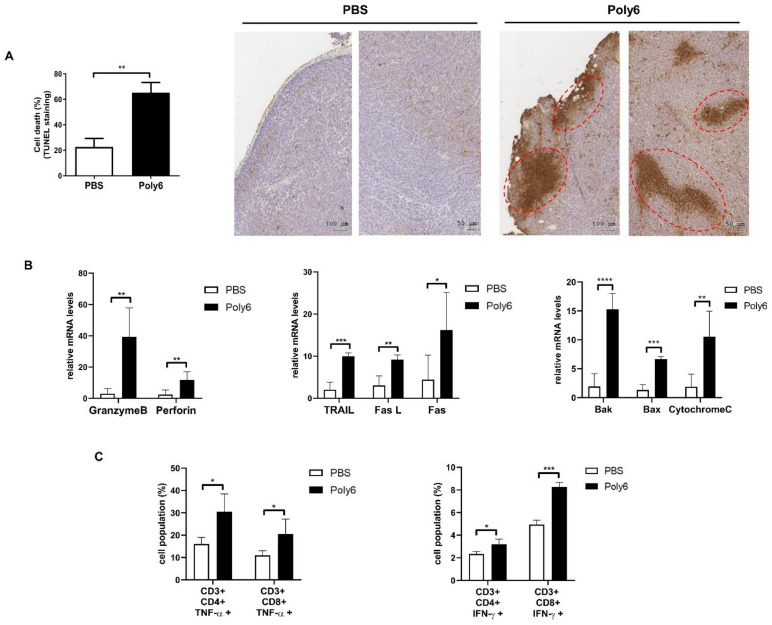
Poly6 exerts anticancer effects via apoptotic tumor cell death in the tumor microenvironment primarily by activating the CD8 T cell-mediated cytotoxic T lymphocyte (CTL) response. (**A**) Tumor tissues were extracted on day 19 after MC38 inoculation. Apoptotic cells in MC38 tumor tissue paraffin sections were identified by terminal deoxynucleotidyl transferase-mediated dUTP nick-end labeling (TUNEL) assay, and apoptotic diaminobenzidine (DAB) positive cells were quantified by tissue FAXS analysis. Increased apoptotic cells indicated by dashed circles. (**B**) Transcription level of death signal-inducing proteins (TRAIL, Fas ligand, Fas), cytolytic proteins (granzyme B, perforin), and pro-apoptotic proteins (Bak, Bax, cytochrome C) in MC38 tumor tissue was assessed by qRT-PCR. (**C**) Populations of TNF-α or IFN-γ producing CD4+ and CD8+ effector T cells in MC38 tumors were analyzed by FACS. Significance differences (* *p* < 0.05, ** *p* < 0.01, *** *p* < 0.001 and **** *p* < 0.0001) among different groups are shown in related figures, and the data are presented as the mean ± standard error of mean (SEM). of the mice (*n* = 4). Student’s *t*-test was used.

Apoptotic cell death of cancer cells in the tumor microenvironment is one of the major features of successful cancer immunotherapy [24]. To examine whether Poly6 treatment would induce apoptosis of cancer cells, tumor sections from mice were analyzed by terminal deoxynucleotidyl transferase-mediated dUTP nick-end labeling (TUNEL) staining. Our data show that Poly6 treatment led to increased apoptotic cell death in cancer tissues (Figure 3A). Furthermore, transcription levels of death signal-inducing proteins (TRAIL), Fas and Fas ligand (FasL), were significantly upregulated in Poly6-treated mice compared to PBS-treated mice. In addition, transcription levels of the well-known cytolytic proteins granzyme B and perforin and pro-apoptotic proteins Bax, Bak, and cytochrome C were also increased in tumor tissues from Poly6-challenged mice (Figure 3B).

Next, we examined the effect of Poly6 treatment on the natural killer (NK) cell or T cell activation responsible for the CTL of cancer cells. In tumor tissue, overall CD4^+^ and CD8^+^ T cell populations were increased in tumor regions of Poly6-treated mice (Figure S5A). Poly6 treatment also significantly increased expression levels of CD44, indicative surface markers for activated T cells, on CD4^+^ and CD8^+^ T cells in the tumor and spleen (Figure S5B,C). We also found that Poly6 treatment increased the production of TNF-α or IFN-γ producing CD4 and CD8 T cells in tumor tissue, which have anticancer effector functions (Figure 3C). However, increased activated T cell populations induced by Poly6 treatment were not found in IFNAR1 KO mice, suggesting that Poly6 leads to enhanced T cell response in an IFN-I-dependent manner (Figure S5D). In addition, the anticancer effects of Poly6 were also observed in melanoma tumor tissue (Figure S5E). However, Poly6 treatment did not lead to significant increases in activated NK cells, the other cells that elicit CTL response, in the MC38-bearing mouse model (Figure S5F). Collectively, our data suggest that the tumor inhibitory effects found in Poly6-treated mice were attributed to tumor cell apoptotic cell death due to activated CD8 T cell-mediated CTL response.

### 2.4. Poly6 Induces Generation of Tip-DCs and CD40 Activation of DCs

**Figure 4:**
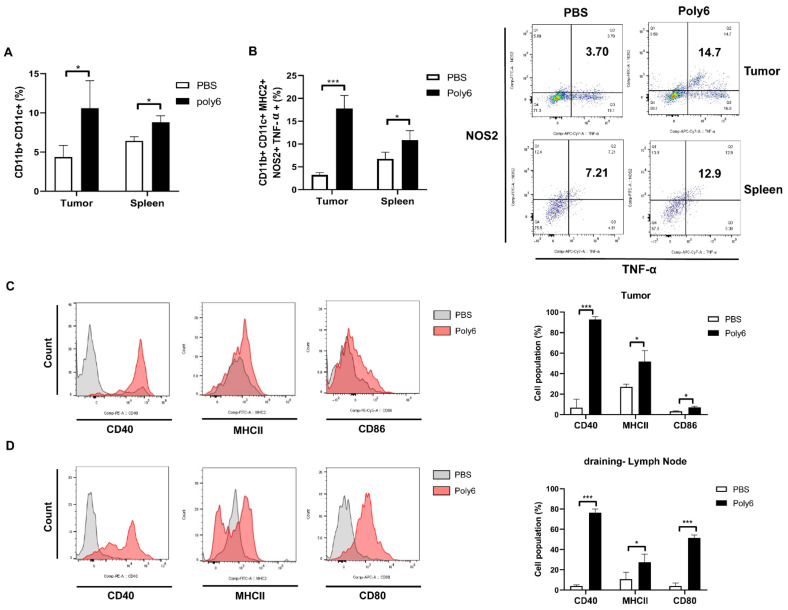
Poly6 induces generation of TNF-a/iNOS-producing DCs (Tip-DCs) and CD40 activation of DCs. (**A**) Populations of CD11b+ CD11c+ dendritic cells in tumor tissue and spleen from MC38-bearing mice were analyzed by FACS. (**B**) Intracellular cytokines (TNF-α+, NOS2+) and surface markers (CD11b+, CD11c+, MHC II +) were stained, and this population was termed Tip-DCs. The population of Tip-DCs in tumor tissues and splenocytes from MC38-bearing mice was analyzed by FACS. (**C**) In tumor tissue and (**D**) draining lymph nodes, maturation markers for dendritic cells were analyzed. CD40, MHC II, and CD86 markers were evaluated in tumors, and CD40, MHC II, and CD80 markers were assessed in the lymph nodes by FACS analysis. Significance differences (* *p* < 0.05 and *** *p* < 0.001) among different groups are shown in related figures, and the data are presented as the mean ± standard error of mean (SEM). of the mice (*n* = 4). Student’s *t*-test were used.

Since Poly6 was subcutaneously injected into a region distant from the site of cancer cell inoculation, and it led to apoptotic cancer cell death primarily due to CD8 T cell-mediated CTL response in the tumor microenvironment, we hypothesized that Poly6 treatment would lead to the recruitment of activated innate APCs, including macrophages or DCs, into the tumor microenvironment, resulting in activation of the cancer-specific CD8 T cell-mediated CTL response. We found that recruitment of DCs (Figure 4A), but not macrophages (Figure S6A), into tumor sites and the spleen was increased, suggesting that DCs may play a major role in inducing cancer-specific T cell responses. Recently, it has been reported that Tip-DCs in the tumor microenvironment, guaranteeing effective CD8 T cells, mediate tumor rejection via CD40 activation [14]. In addition, our data demonstrate that Poly6 treatment, as shown in Figure 1, leads to enhanced IFN-I production in DCs, resulting in enhanced production of Tip-DCs. Interestingly, we found that Poly6 treatment in MC38-bearing mice led to an increased population of Tip-DCs in the tumor region and spleen via a gating strategy (Figure 4B and Figure S6B). A similar trend was also observed in tumor tissue from B16F10-bearing mice (Figure S6C). In addition, we found that Tip-DC induced by Poly6 treatment was inhibited in IFNAR1 KO mice, further supporting our finding (Figure 1G) that Tip-DC by Poly6 is dependent on IFN-I production (Figure S6D). Poly6 treatment also enhanced expression of DC maturation markers CD40, CD80, CD86, and MHC class II molecules in tumor and draining lymph node tissues in MC38-bearing mice (Figure 4C,D). In particular, for maturation markers, CD40 expression in DCs was dramatically induced in both cancer tissue and spleen from Poly6 challenged mice. Our data suggest that Poly6 treatment enhances the production of Tip-DCs and their recruitment into the tumor microenvironment, resulting in effective CD8 T cell-mediated tumor rejection, likely via CD40 activation. Next, we checked the transcription level of IL-12 and CC chemokine receptor type 2 (CCR2), markers of Tip-DC development in cancer tumor tissue via RT-qPCR. Our data show that Poly6 versus PBS treatment led to enhanced transcription levels of IL-12 and CCR2 in tumor tissues (Figure S7A,B).

### 2.5. Poly6 Leads to Direct Oncolytic Activity of Tip-DCs in an NO-Dependent Manner

**Figure 5:**
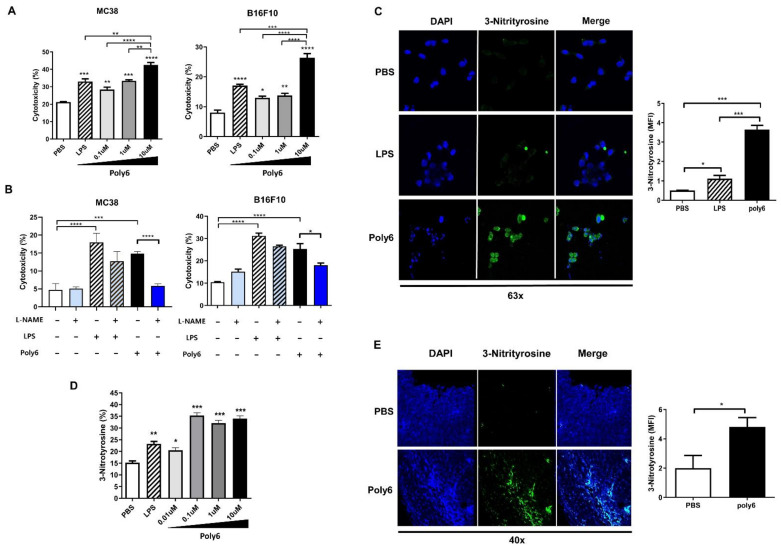
Poly6 leads to direct oncolytic activity of Tip-DCs in an NO-dependent manner. (**A**) Cancer cells (MC38 colon cancer, B16F10 melanoma cancer) were analyzed using a co-culture system with Tip-DCs generated by Poly6. DC2.4 cells were treated with Poly6 for 48 h, and CFSE-labeled cancer cells were co-cultured with Poly6 stimulated DC2.4 cells for 4 h. Then, dead cancer cells were evaluated by 7AAD positive staining and analyzed by FACS; *n* = 3 biologically independent samples. (**B**) Inhibited cytotoxicity of cancer cells by addition of L-NAME was evaluated by FACS. DC2.4 cells were treated with Poly6 (10 µM) and/or L-NAME (5 mM) for 48 h. Poly6 stimulated DC2.4 cells and CFSE labeled cancer cells were co-cultured. 7AAD-positive and CFSE-labeled cancer cells were evaluated as an oncolytic response; *n* = 3 biologically independent samples. (**C**) Peroxynitrite levels were evaluated by assessing 3-nitrotyrosine levels. Supernatants of DC2.4 cells treated with Poly6 (1 µM) for 48 h were treated with MC38 cancer cells for 4 h. Then, cancer cells were permeabilized and stained with 3-nitrotyrosine antibody. Images were analyzed by confocal microscopy; *n* = 3 biologically independent samples. (**D**) 3-Nitrotyrosine levels were analyzed by FACS; *n* = 4 biologically independent samples. (**E**) Peroxynitrite in tumor paraffin sections was evaluated by 3-nitrotyrosine staining and analyzed by confocal microscopy (*n* = 4). These results are representative of two independent experiments. Significance differences (* *p* < 0.05, ** *p* < 0.01, *** *p* < 0.001 and **** *p* < 0.0001) among different groups are shown in related figures, and the data are presented as the mean ± standard error of mean (SEM). Student’s *t*-test, one- and two-way ANOVA were used.

Previously, it was reported that innate APCs exert anticancer effects via an iNOS-mediated NO-dependent mechanism [25,26,27]. Furthermore, we found that Poly6 treatment also attenuates cancer growth and weight, even in nude mice challenged with HBV W4P large surface protein-expressing NIH-3T3 cells (Figure S8A,B) [28], suggesting that innate immune cells could inhibit tumor progression independent of T cells. Therefore, we examined the direct oncolytic activity of Tip-DCs induced by Poly6 treatment in various cancer cell lines. To this end, Tip-DC-mediated oncolytic activity was evaluated using a co-culture system between Poly6-stimulated DC2.4 cells and carboxyfluorescein succinimidyl ester (CFSE)-labeled MC38 or B16F10 cells. We found that Poly6 treatment led to increased death of cancer cells in a dose-dependent manner (Figure 5A). Furthermore, we investigated the oncolytic potential of Poly6 treatment in various cancer cell lines using the co-culture system. We found that Poly6-treated DCs also mediated enhanced oncolytic activity in E0771 mouse breast cancer cells, PanO2 mouse pancreatic cancer cells, and MDA231 human breast cancer cells (Figure S8C).

Next, we explored whether Poly6-treated DCs would induce direct tumor killing via an NO-dependent mechanism. To this end, we investigated the inhibitory effect of an iNOS inhibitor, N omega-Nitro-L-arginine methyl ester hydrochloride (L-NAME), on the oncolytic activity of Poly6. We found that the addition of L-NAME to Poly6-treated DC2.4 cells led to the inhibition of the oncolytic activity of Poly6 in various cancer cell lines, including MC38, B16F10, and EO771 (Figure 5B and Figure S8D), suggesting that Poly6-treated DCs elicit anticancer effects via the iNOS-NO axis. Also, the oncolytic effect of Poly6 in a cancer implanted mouse model was inhibited in L-NAME treated mice (Figure S9A–D). Moreover, the Tip-DC generation and T cell activation in tumor tissue by Poly6 were also inhibited in L-NAME-treated mice (Figure S9E,F). In addition, we also checked whether Poly6 treatment would lead to generation of an NO-derived oxygen radical, peroxynitrite, known to elicit strong anticancer effects via apoptotic cancer cell death [29,30]. We found—by immunostaining of nitrotyrosine—an indicator of peroxynitrite formation, that is, that Poly6-treated DC2.4 supernatant mediated enhanced intracellular peroxynitrite accumulation within MC38 cancer cells, resulting in shrunken nuclei, a feature of apoptotic cell death, even more so than LPS treated DC2.4 cells (Figure 5C,D). Furthermore, we also found enhanced peroxynitrite accumulation in the tumor tissues of Poly6-treated MC38-bearing mice (Figure 5E). Taken together, our data suggest that Tip-DCs induced by Poly6 exert an anticancer effect by direct oncolytic activity via iNOS-dependent production of NO or peroxynitrite.

### 2.6. Combination of Poly6 with Anti-PD-L1 Ab Treatment Exerts an Enhanced Anticancer Effect in Mice

**Figure 6:**
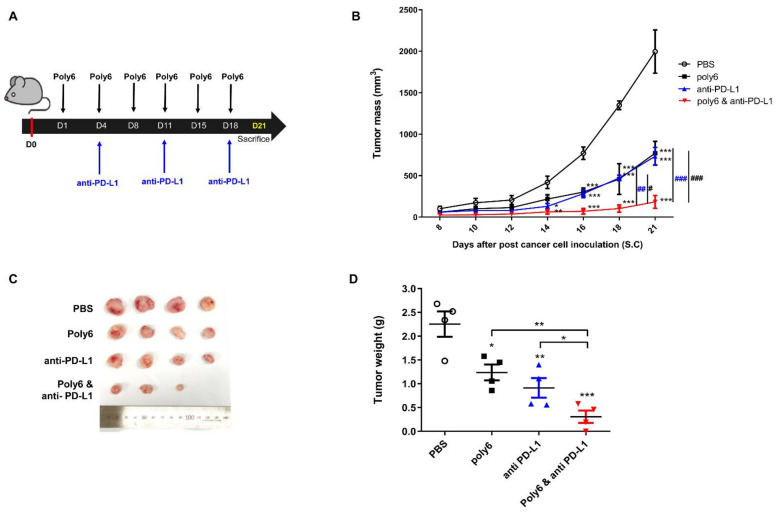
Combination of Poly6 with anti-PD-L1 Ab treatment exerts enhanced anticancer effects in a cancer bearing mouse model. (**A**) Schematic combination therapy in vivo experiment schedule. Poly6 peptide (10 μg) was subcutaneously injected, and anti-PD-L1 antibody (100 μg) was intraperitoneally injected following the injection schedule. MC38 cancer cells (1 × 10^6^ cells/100 µL) were inoculated on day 0 in C57BL/6 mice (*n* = 4). (**B**) Tumor growth was observed for 21 days. (**C**) Tumor tissue image on day 21. (**D**) Weight of tumor tissue was compared. These results are representative of two independent experiments. Significance differences (* *p* < 0.05, ** *p* < 0.01 and *** *p* < 0.001) among different groups are shown in related figures, and the other significance differences (# *p* < 0.05; ## *p* < 0.01 and ### *p* < 0.001) are used to compare with the Poly6 and anti-PD-L1 combination group. The data are presented as the mean ± standard error of mean (SEM). of the mice (*n* = 4). Student’s *t*-test, one- and two-way ANOVA were used.

Our data indicate that Poly6 exerts a therapeutic anticancer effect via apoptotic cancer cell death by Tip-DC-mediated NO-dependent direct killing and indirect killing via an enhanced CTL response. Combination treatment of CD40-activating agents with immune checkpoint inhibitor agents has been reported to have an enhanced anticancer effect [31]. Therefore, we sought to evaluate the additive anticancer effect of Poly6 treatment with anti-PD-L1 Ab, an immune checkpoint inhibitor [32], in the MC38-bearing mouse model (Figure 6A). Combination treatment with Poly6 and anti-PD-L1 demonstrated a significant reduction in tumor growth from 18 days onward compared to single treatment with Poly6 or anti-PD-L1 (Figure 6B,C). In addition, after mice were sacrificed, tumor weight was found to be significantly reduced in mice subjected to combination treatment compared to mice given single treatment (Figure 6D). Moreover, we found an increased population of activated CD44^+^ T cells in tumor (Figure S10A) and TNF-α producing effector CD4^+^ and CD8^+^T cells in the spleen (Figure S10B). We also found that combination treatment significantly increased FasL mRNA in tumor tissue compared to PBS or single treatment (Figure S10C). Taken together, our data suggest that Poly6 treatment exerts enhanced anticancer effects with anti-PD-L1 Ab treatment in a implanted cancer mouse model.

## 3. Discussion

A DC subset termed Tip-DC has been reported to play a pivotal role in controlling or providing immunity to pathogenesis in several types of infectious diseases, including *Trypanosoma brucei brucei* [33] and *Listeria monocytogenes* infections [34]. In addition, the novel therapeutic potential of Tip-DCs in the cancer field has recently been described [17]. They contribute to cancer inhibition primarily via the CD8 T cell-mediated CTL response by activation of CD40 in an NO-dependent manner, suggesting that introducing a new agent favoring Tip-DC development may a be feasible option for cancer immunotherapy.

In this study, we examined the potential of Poly6, an HBV-derived 6-mer peptide [15], as a new immune-modulating anticancer drug that inhibits tumor progression via enhanced production of Tip-DCs. First, we found that Poly6 treatment, capable of exerting anti-HIV-1 effects, leads to IFN-I production in DCs, the DC2.4 cell line, and BMDCs via mitochondrial stress-mediated cytosolic exposure of mitochondrial DNA (Figure 1B and Figure S1C–E). Increasing evidence has shown that various drugs targeting molecules that modulate host acetylation status, such as histone acetylation transferases (HATs) or histone deacetylases (HDACs), affect mitochondrial homeostasis or metabolism [19,35,36] or enhance IFN-I production via acetylation modification of phosphorylated-signal transducer and activator of transcription 1 (p-STAT-1) [37] or IFN regulatory factor 3 (IRF-3) [38]. However, the links between cellular modulation and induced IFN-I production found in Poly6-treated DCs require further elucidation in future studies. Enhanced IFN-I production in DCs has been reported as a signature of Tip-DCs [16], which can lead to enhanced TNF and iNOS-mediated NO production or DC maturation, resulting in harnessing T cell-mediated immune responses [14]. Actually, our in vitro experiments demonstrated that Poly6 treatment leads to an increased Tip-DC population and maturation of DC cells in an IFN-I-dependent manner (Figure 1A,G), resulting in direct oncolytic activity in various cancer cell lines via iNOS-mediated NO and peroxynitrite production (Figure 5). Furthermore, Poly6 vaccination via subcutaneous injection in cancer-bearing mice enhanced Tip-DC production and their recruitment into the tumor microenvironment (Figure 4), resulting in direct cancer cell death via NO or peroxynitrite production and indirect inhibition of tumor progression via the CD8 T cell-mediated CTL response (Figure 3C and Figure 5). It has also been reported that the enhanced CD8 T cell-mediated CTL response induced by Tip-DCs is primarily due to augmentation of the CD40-CD40L pathway [14]. Consistently, our in vivo data show that Poly6 vaccination also led to a strong enhancement of CD40 expression in tumor infiltrated DCs (Figure 4C) compared to other DC costimulatory molecules (CD80, CD86, and MHC class II).

Generally, the CD40-CD40L interaction between CD4+ T cells and dendritic cells (DCs) primes DCs to activate CD8+ T cell-mediated CTL response [39,40,41]. Therefore, CD40-CD40L also represents an attractive target pathway for cancer immunotherapy. Many cancer immunotherapies using CD40 agonists to therapeutically activate DCs and other myeloid cells have been developed to date [42,43]. Since single-agent immunotherapy is generally ineffective for the majority of patients with advanced cancers [44], the combined approach of multiple therapeutic agents is implemented to achieve complete remission and cures. Particularly, CD40 agonists enhance anticancer immune responses with several types of immune checkpoint inhibitors [31]. Consistent with this notion, our data demonstrate that Poly6, a new CD40 inducer, also leads to an enhanced cancer inhibition with anti PD-L1 Ab (Figure 6 and Figure S10).

Of note, it has also been reported that increased CD40 expression can lead to enhanced IFN-I production via the STING-dependent axis [45]. Therefore, despite still not being proven, it is likely that the enhanced IFN-I production observed in Poly6-treated DCs may be due to enhanced CD40 expression. However, the relationships between CD40, IFN-I, and Poly6 treatment in Tip-DC need to be elucidated in the future.

Poly6 could induce anticancer effects via DC-produced NO dependent manner in vivo. However, in this study, we challenged mice with Poly6 via a subcutaneous route, not an intravenous route, in a separate region from the site of cancer cell inoculation, suggesting that the anticancer effects of Poly6 observed in this study were achieved only via DC-mediated immune response. Hence, Poly6 injection via the subcutaneous route seems to minimize the risk of side effects due to the injection. Furthermore, Poly6 is capable of inducing DC activation and maturation, providing a rationale for its use as an adjuvant to several vaccine modules, including protein-based subunit or DNA vaccine, or cancer immunotherapy. This possibility will be addressed in the future.

Our present study has several limitations. Our data could not show a dose dependent effect of Poly6 on BMDC maturation, despite a very broad (pico- to micromolar) concentration range of Poly6 treatment (Figure 1B–F). In addition, there were also differences between iNOS expression in Poly6-treated BMDC (Figure 1E) and the nitrate concentration in the same cells (Figure 1F). Given that mitochondrial DNA stress induced by Poly6 treatment could affect diverse biological signaling, it may be due to the convergence effects of several distinct pathways induced by Poly6. So, to address this issue, the exact signaling pathway of Poly6 in DCs should be elucidated in the future.

In conclusion, our data reveal that Poly6 treatment elicits a strong antitumor immune response in mice, possibly via an IFN-I-dependent Tip-DC-inducing capacity, contributing to tumor clearance in two ways: direct cancer cell killing by Tip-DCs, which is iNOS-dependent and involves NO and peroxynitrite production, and indirect killing by CD8+ T cell-mediated CTL response via a CD40 activation, suggesting the potential use of Poly6 as an adjunct immunotherapy that can enhance the effect of immune checkpoints.

## 4. Materials and Methods

### 4.1. Mice

Six-week-old female C57BL/6 mice, Balb nu/nu mice, and IFNAR1 knockout mice were purchased from Orient Bio (Orient Bio Inc., Seongnam, Korea) and maintained in a specific pathogen-free (SPF) environment. All procedures were approved in advance by the Institutional Animal Care and Use Committee of Seoul National University (SNU-181010-2).

### 4.2. Cells and Cell Culture

Murine MC38 colon cancer cells engineered to express human carcinoembryonic antigen cells (MC38/CEA) (Kerafast, Boston, MA, USA), murine breast cancer cells (EO771) (ATCC, Manassas, USA), murine pancreatic cancer cells (PanO2) (ATCC, Manassas, USA), and HBV W4P large surface protein-expressing NIH-3T3 cells (W4P-LHB-NIH3T3) [28] were cultured in Dulbecco’s modified Eagle’s medium (DMEM), and human breast cancer cells (MCF-7, MDA231) (ATCC, Manassas, USA), mouse melanoma cells (B16F10) (ATCC, Manassas, USA), and DC2.4 cells (Huiying Bio. Tech, Shanghai, China) were cultured in Roswell Park Memorial Institute 1640 Medium (RPMI). These media were supplemented with 10% fetal bovine serum (FBS), 100 U/mL penicillin, and 100 μg/mL streptomycin in an incubator at 5% CO_2_ and 37 °C. Bone marrow-derived dendritic cells (BMDCs) were harvested from the bone marrow of 7-week-old C57BL/6 mice and were differentiated for 5 days in Iscove’s Modified Dulbecco’s Medium (IMDM) supplemented with recombinant mouse granulocyte-macrophage colony stimulating factor (GM-CSF) (20 ng/mL), mouse IL-4 (20 ng/mL), 10% FBS, penicillin (100 U/mL), streptomycin (100 μg/mL), gentamicin (50 μg/mL), L-glutamine (2 mM), and β-mercaptoethanol (50 nM) [46].

### 4.3. Tumorigenesis Studies

MC38 colon cancer cells engineered to express human carcinoembryonic antigen (MC38/CEA) (1 × 10^6^/100 µL) (Kerafast, Boston, MA, USA), B16F10 melanoma cancer cells (1 × 10^6^/100 µL) (ATCC, Manassas, USA), and PanO2 pancreatic cancer cells (1 × 10^6^/100 µL) (ATCC, Manassas, USA) were injected subcutaneously into the right flank of each mouse. In this study, MC38 cells refer to MC38/CEA cancer cells. The Poly6 peptide (10 µg/100 µL) was injected subcutaneously separately from the tumor injection site. For combined therapeutic effect, anti-PD-L1 (100 μg) was administered intraperitoneally. In the nude mouse experiments, W4P-LHB-NIH3T3 cells (1 × 10^8^ cells/100 µL) were injected subcutaneously, and cisplatin (50 µg) was injected peritumorally as previously described [28]. To prove the inhibition effect of NO in the anticancer effect of Poly6 in the mouse model, L-NAME (2 mg/100 µL) was administrated intravenously 3 times before MC38 cell inoculation; post cancer inoculation, L-NAME treatment was given 3 more times. Tumor mass was measured and calculated with the following formula: width × width × length × 0.52. All mice were sacrificed by CO_2_ asphyxiation, and then tissues were dissected for subsequent experiments.

### 4.4. Histopathological Study

Fixed tumor tissues were embedded in paraffin and sectioned to a thickness of 5 μm. Sectioned slides were stained with hematoxylin and eosin (H&E). Slides were analyzed with the Aperio Scanscope (Leica Microsystems, IL, USA), and quantification of stained antigens was analyzed using HistoQuest (Tissue-Gnostics^®^, Vienna, Austria). Apoptotic cell death in tumor tissues was detected by terminal deoxynucleotidyl transferase-mediated dUTP nick-end labeling (TUNEL) assay using the ApopTag Peroxidase In Situ Apoptosis Detection Kit (Millipore, MA, USA). Stained apoptotic cells were digitalized by the Aperio Scanscope and HistoQuest.

### 4.5. Flow Cytometry

Tumors were dissociated with 200 U/mL collagenase IV (Sigma-Aldrich, St. Louis, MO, USA) and 200 μg/mL DNase I (Sigma-Aldrich, St. Louis, MO, USA) at 37 °C for 30 min with shaking. For flow cytometry, cells were blocked with CD16/32 (#101301, BioLegend, San Diego, CA, USA). For intracellular cytokine staining, cells were fixed in 1% paraformaldehyde in PBS and permeabilized using 0.1% Triton X-100 in FACS buffer containing 10% FBS and 10 mM ethylenediaminetetraacetic acid (EDTA) in PBS for 20 min [47]. The following antibodies were used: anti-CD3 (17A2), anti-CD4 (RM4-5), anti-CD8 (53–6.7), anti-CD11b (M1/70), anti-CD11c (HL3), anti-CD40 (3/23), anti-CD80(B7-1), anti-CD86 (GL-1), anti-CD44 (IM7), anti-CD25(3C7), anti-TNF-α (MP6-XT22), anti-iNOS (CXNFT), anti-IFN-γ (XMG1.2), and 3-nitrotyrosine (39B6). All antibodies were purchased from BioLegend, BD Bioscience (San Jose, CA, USA), and eBioscience (San Diego, CA, USA).

### 4.6. Analysis of mRNA by Real-Time PCR

Total mRNA was extracted from tumor tissues and cells using TRIzol reagent and quantified. Relative expression levels were determined using the SYBR green kit (#74005, Bioline, London, UK). Primer sequences are shown in Table S1.

### 4.7. Western Blot

Harvested cells were lysed using radioimmunoprecipitation assay buffer (RIPA buffer) (#9806, Cell Signaling Technology, Danvers, MA, USA) with phosphate inhibitor and protease inhibitor (Hoffmann-La Roche Inc., Basel, Switzerland). After protein quantification by Bradford assay and denaturation by boiling, protein samples were separated by electrophoresis, transferred to nitrocellulose (NC) membranes, and blocked for 1 h. Membranes were incubated overnight at 4 °C with primary antibodies (1:1000). Primary antibodies used were as follows: P300 (#sc-8981, Santa Cruz Biotechnology, Dallas, TX, USA), NOS2 (#sc-4271, Santa Cruz Biotechnology), cGAS (#31659, Cell Signaling Technology), and STING (#13647, Cell Signaling Technology). The next day, membranes were incubated with horseradish peroxidase (HRP)-conjugated secondary antibodies (1:2000) for 2 h. After enhanced chemiluminescence (ECL) solution was applied to the membranes, proteins were detected on an ImageQuant LAS 2000 (GE Healthcare, Chicago, IL, USA).

### 4.8. Immunofluorescence

Cells were seeded on two-chamber glass slides (Nunc, Roskilde, Denmark). After cells were attached, they were washed with PBS. Cells were incubated in RPMI containing 1% FBS in the presence of PBS, LPS, or Poly6 for 12 h. Cells were then fixed in 4% paraformaldehyde solution for 10 min and permeabilized with 0.1% Triton-X 100 for 10 min. Cells were blocked for 1 h and stained with 3-nitrotyrosine [25] for 10 min. Nuclear staining of cells was performed with diamidino-2-phenylindole (DAPI) mounting medium (VECTASHIELD Antifade Mounting Medium, #H-1000) (Vector Laboratories, Burlingame, CA, USA). Images were obtained using a confocal microscope (Leica STED CW) [20].

### 4.9. Cytotoxicity

#### 4.9.1. Direct Cell Cytotoxicity

Cancer cells were seeded (2 × 10^5^ cells) on 96-well microplates and incubated with increasing concentrations of Poly6 for 24 h. Triton X 100 (0.1%) was used as a positive control. CytoTox 96^®^ Reagent (Promega, Madison, WI, USA), which measures lactate dehydrogenase (LDH), and an equal volume of culture supernatants were added and incubated for 30 min. The absorbance was measured at 490 nm.

#### 4.9.2. Cell-Mediated Cytotoxicity

DC2.4 cells were seeded (2.5 × 10^5^ cells) on 96-well microplates and incubated with increasing concentrations of Poly6 for 48 h. Cancer cells (MC38, B16F10, EO771l, PanO2, MDA231) were stained with CFSE for 10 min. CFSE-labeled cells were co-cultured with DC2.4 cells, for which the effector target ratio was 5:1. CFSE-labeled cancer cell death was estimated by 7AAD staining using FACS analysis.

### 4.10. Cytokine and Nitrate Assay

DC2.4 cells were seeded (1 × 10^6^ cells) on 6-well plates for 12 h. Cells were then starved with opti-MEM for 1 h. After starvation, cells were cultivated with a gradient of Poly6, PBS, or LPS for 48 h. Culture supernatants were used to measure cytokine levels. To assess cytokine production by DC2.4 cells, interferon beta and TNF-α were measured using enzyme-linked immunosorbent assay kits (ELISA, eBioscience). Nitric oxide was measured using a nitrite/nitrate assay kit (Sigma) according to the manufacturer’s protocol.

### 4.11. Dissociation of Tumor, Lymph Nodes, and Spleen

Tumors and lymph nodes were excised and digested in collagenase IV (200 U/mL, Sigma) and DNase I (200 μg/mL, Sigma) in 1% FBS RPMI media for 30 min at 37 °C with shaking. Next, EDTA was added, and the supernatant and cells were filtered through a 70 μm filter. The spleen was excised and filtered through a 70 μm filter as well. After centrifugation at 1500 rpm for 5 min, red blood cell (RBC) lysis buffer (Sigma) was added for 3 min. Next, 10% FBS containing RPMI was added and the suspended cells were centrifuged at 1500 rpm for 5 min. Pellets were resuspended in complete 10% FBS containing RPMI [48].

### 4.12. Measurement and Quantification of Oxidative DNA Damage

An 8-OHdG competitive ELISA was performed using a commercial kit (OxiSelect Oxidative DNA Damage ELISA Kit (8-OHdG Quantitation)) (Cell Biolabs, San Diego, CA, USA) according to the manufacturer’s instructions.

### 4.13. Detection of Mitochondrial ROS and Cytosolic Mitochondrial DNA

#### 4.13.1. Mitochondrial ROS

Cells were stained with mitoSOX, a mitochondrial superoxide indicator (Invitrogen, CA, USA) to evaluate mitochondrial ROS. Cells were cultured in 96-well flat-bottom plates for FACS analysis, and on two-chamber glass slides (Nunc, Roskilde, Denmark) for immunofluorescence. Adherent cells were stimulated by PBS (control) or Poly6 (10 µM). Stimulated cells were stained with mitoSOX for 20 min at 37 °C. MitoSOX intensity levels in cells were measured by FACS and confocal microscopy.

#### 4.13.2. Cytosolic Mitochondrial DNA

Cells were stimulated with Poly6 for 24 h. The cytosolic fraction was isolated using the Qproteome Cell Compartment Kit (Qiagen, Hilden, Germany) according to the manufacturer’s protocol. Cell pellets were resuspended in 4 °C PBS and centrifuged at 500× *g* for 10 min. The supernatant was removed and pellets were resuspended in protease inhibitor solution containing lysis buffer and then incubated at 4 °C for 10 min. Lysates were centrifuged at 1000× *g* for 10 min. After centrifugation, supernatants were transferred to new tubes. Supernatants were subjected to the PCI method to extract DNA. Mitochondrial DNA was detected using the primer sets listed in Table S1.

### 4.14. Statistical Analysis

Statistical comparison of results was performed by *t*-test, as well as one and two-way ANOVA. Data are shown as the mean ± standard error of mean (SEM) and were analyzed using GraphPad Prism version 8.0 (GraphPad, La Jolla, CA, USA). Statistical significance is denoted with asterisks as follows: * *p* ≤ 0.05; ** *p* ≤ 0.01; *** *p* ≤ 0.001; and **** *p* ≤ 0.0001.

## 5. Conclusions

In conclusion, our findings show that Tip-DCs induced by Poly6 exert anticancer responses in the tumor microenvironment in two ways. On one hand, Tip-DCs exert direct iNOS/NO-mediated oncolytic activity by generating an NO-derived oxygen radical, peroxynitrite. On the other hand, CD8+ T cells activated by Tip-DCs, via activated CD40, can induce tumor progress suppression via cytotoxic T lymphocyte responses. Furthermore, improved anticancer effects can be expected by adding Poly6 to anti-PD-L1, an immune checkpoint inhibitor. Our findings could potentially open up a new avenue for anticancer immunotherapy.

## Data Availability

The data presented in this study are available on request from the corresponding author.

## Supplementary Materials

The following are available online at https://www.mdpi.com/2072-6694/13/3/407/s1, Figure S1: Poly6 treatment leads to Tip-DCs development via mitochondrial DNA stress and cyclic GMP-AMP synthase-stimulator of interferon genes (cGAS–STING) axis, Figure S2: Poly6 treatment leads to enhanced IL-12 production from DCs in an IFN independent manner, Figure S3: Poly6 treatment cannot lead to direct anticancer effect on various cancer cells, Figure S4: Poly6 exerts anticancer effects on a pancreatic and pre-activation cancer mouse model, Figure S5: Poly6 exerts anticancer effects via induction of T cell activation in the tumor microenvironment, Figure S6: Poly6 induces the generation of Tip-DCs in tumor tissue from a cancer-bearing mouse model, Figure S7: Poly6 treatment can lead to enhanced transcription of IL-12 and CCR2 in tumor tissues, Figure S8: Poly6 induces direct oncolytic activity of Tip-DCs in an NO-dependent manner, Figure S9: Inhibition of anticancer effect of Poly6 in a tumor-bearing mouse model by L-NAME treatment, Figure S10: Combination of Poly6 with anti-PD-L1 Ab treatment exerts an enhanced anticancer effect by inducing T cell activation, Figures S11–S14: The whole Western blot figures and intensity ratio of each band, normalized by Glyceraldehyde 3-phosphate dehydrogenase (GAPDH), Table S1: Primers used for qPCR.
