# Supplementary material for: A Hepatitis B Virus-Derived Peptide Exerts an Anticancer Effect via TNF/iNOS-producing Dendritic Cells in Tumor-Bearing Mouse Model
Source: Cancers (Basel). 2021 Jan 22;13(3):407. doi: 10.3390/cancers13030407 (PMC7865762; doi:10.3390/cancers13030407)
Supplement: Supplementary file 1 [file cancers-13-00407-s001.pdf]

Supplementary Files

A Hepatitis B Virus-Derived Peptide Exerts an Anticancer Effect via TNF/iNOS-producing Dendritic Cells in Tumor-Bearing Mouse Model

Soo-Bin Yang, Mi-Hyun Lee, Bo-Ram Kim, Yu-Min Choi and Bum-Joon Kim

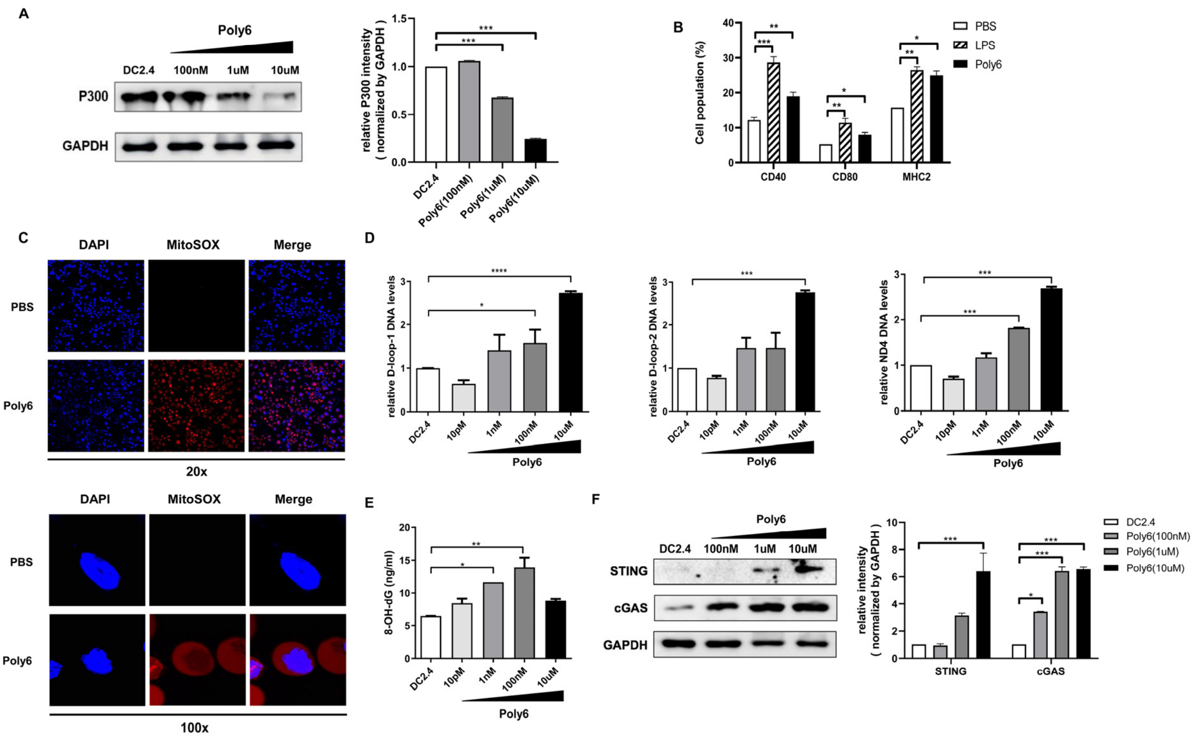

Figure S1. Poly6 treatment leads to Tip-DCs development via mitochondrial DNA stress and cyclic GMP-AMP synthase-stimulator of interferon genes (cGAS–STING) axis**.** (**A**) DC2.4 cells were incubated with Poly6 for 24 h. P300 protein levels were evaluated by Western blotting. (**B**) DC2.4 cells were treated Poly6 (10 µM) for 24 h. LPS was used as a positive control. Maturation of DC2.4 cells treated with Poly6 was assessed by flow cytometry. (**C**) DC2.4 cells were incubated with Poly6 (10 µM) for 12 h. Cells were stained for mitochondrial superoxide (MitoSOX, red) and nuclei (DAPI, blue) and then analyzed using confocal microscopy at 20× and 100× magnification. (**D**) Cytosolic mitochondrial DNA was extracted from nuclear and cytosolic fractions of DC2.4 cells that were treated with Poly6 for 24 h. Measurement of cytosolic mitochondrial DNA expression by qRT-PCR using the mitochondrial D-loop-1, D-loop-2 and ND4 primer sets. (**E**) Cytosolic DNA was obtained from Poly6 stimulated DC2.4 cells, and levels of 8-OHdG were measured using an ELISA kit. (**F**) STING and cGAS protein levels were assessed by Western blotting assay and detected by LAS 2000. These results are representative of two independent experiments. Significance differences (* $p < 0.05$, ** $p < 0.01$, *** $p < 0.001$ and **** $p < 0.0001$) among different groups are shown in related figures, and the data are presented as mean ± s.e.m.; $n = 3$ biologically independent samples. Student's t-test was used.

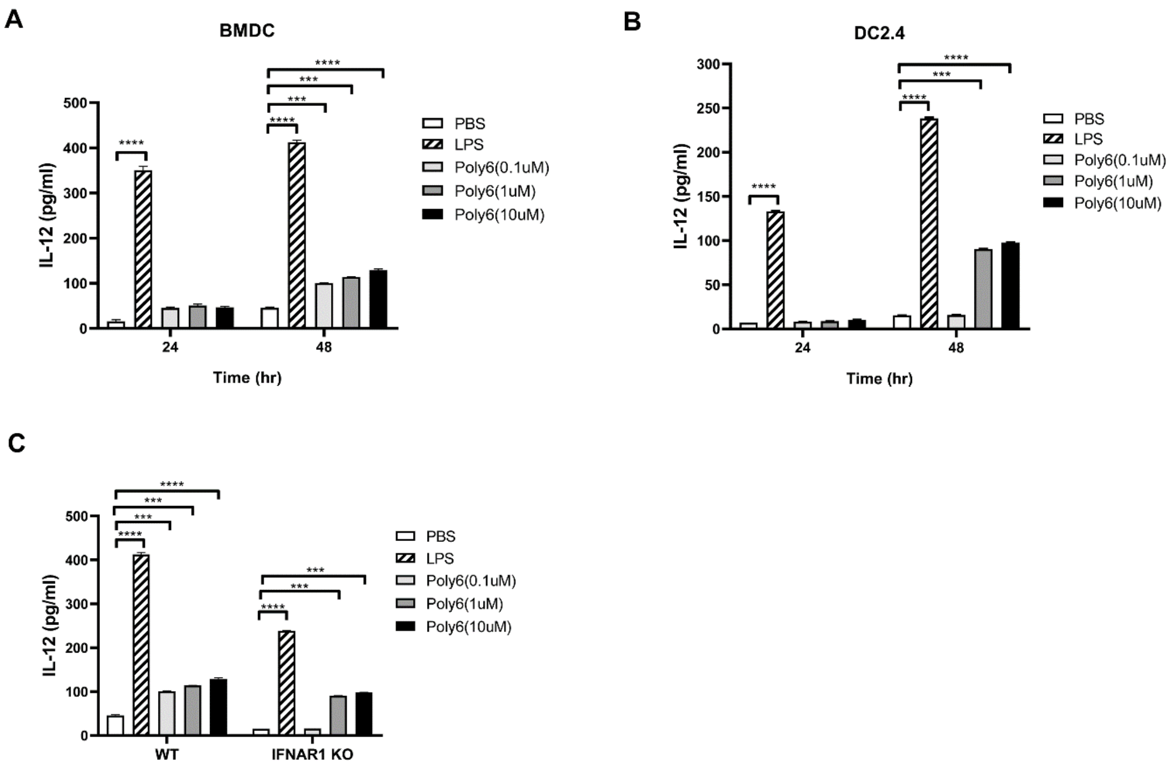

Figure S2. Poly6 treatment leads to enhanced IL-12 production from DCs in an IFN independent manner**.** DC2.4 cells (**A**) and BMDCs (**B**) from wild type mice were incubated with Poly6 for 24 h or 48 h. LPS (1 μg/mL) was used as a positive control. Culture supernatants were used to measure IL-12. (**C**) BMDCs from wild-type mice and IFNAR1 KO mice were treated Poly6 for 48 h. IL-12 production was measured by ELISA Kit. Significance differences (* $p < 0.05$, ** $p < 0.01$, *** $p < 0.001$ and **** $p < 0.0001$) among different groups are shown in related figures, and the data are presented as mean ± s.e.m.; $n = 3$ biologically independent samples. Student's t-test, two- way ANOVA were used.

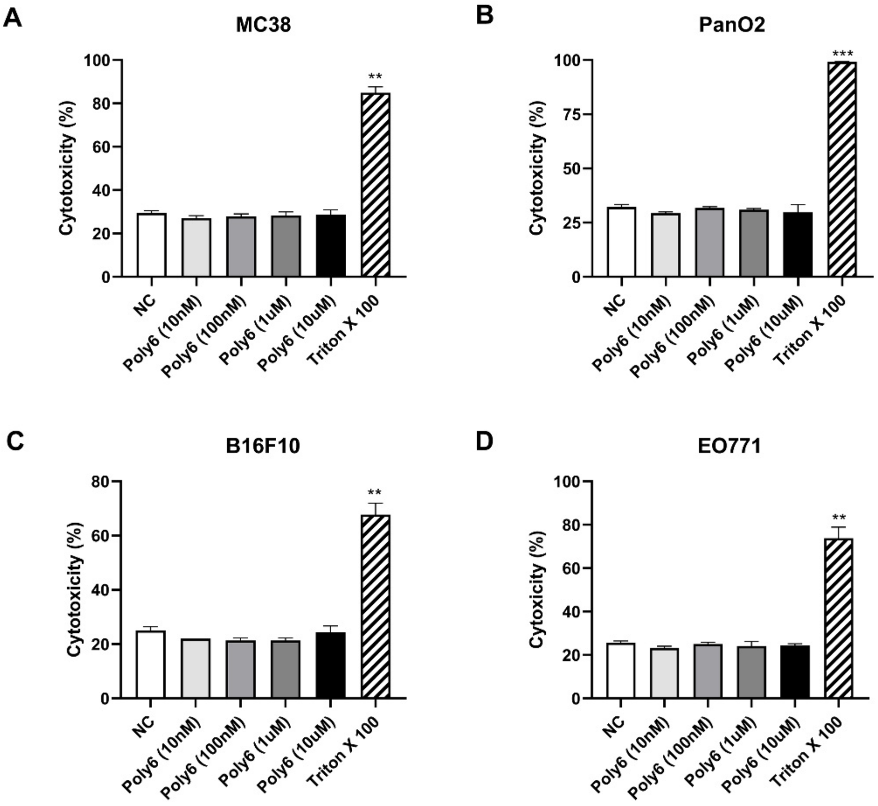

Figure S3. Poly6 treatment cannot lead to direct anticancer effect on various cancer cells. MC38 (**A**), PanO2 (**B**), B16F10 (**C**) or EO771 (**D**) cancer cells (2×10^5 cells) were seeded on 96-well microplates and incubated with increasing concentrations of Poly6 for 24 h. Triton X 100 (0.1%) was used as positive control. Culture supernatants were used to measure lactate dehydrogenase (LDH). Significance differences (* $p < 0.05$, ** $p < 0.01$, *** $p < 0.001$ and **** $p < 0.0001$) among different groups are shown in related figures, and the data are presented as mean ± s.e.m.; $n = 4$ biologically independent samples. Student's t-test was used.

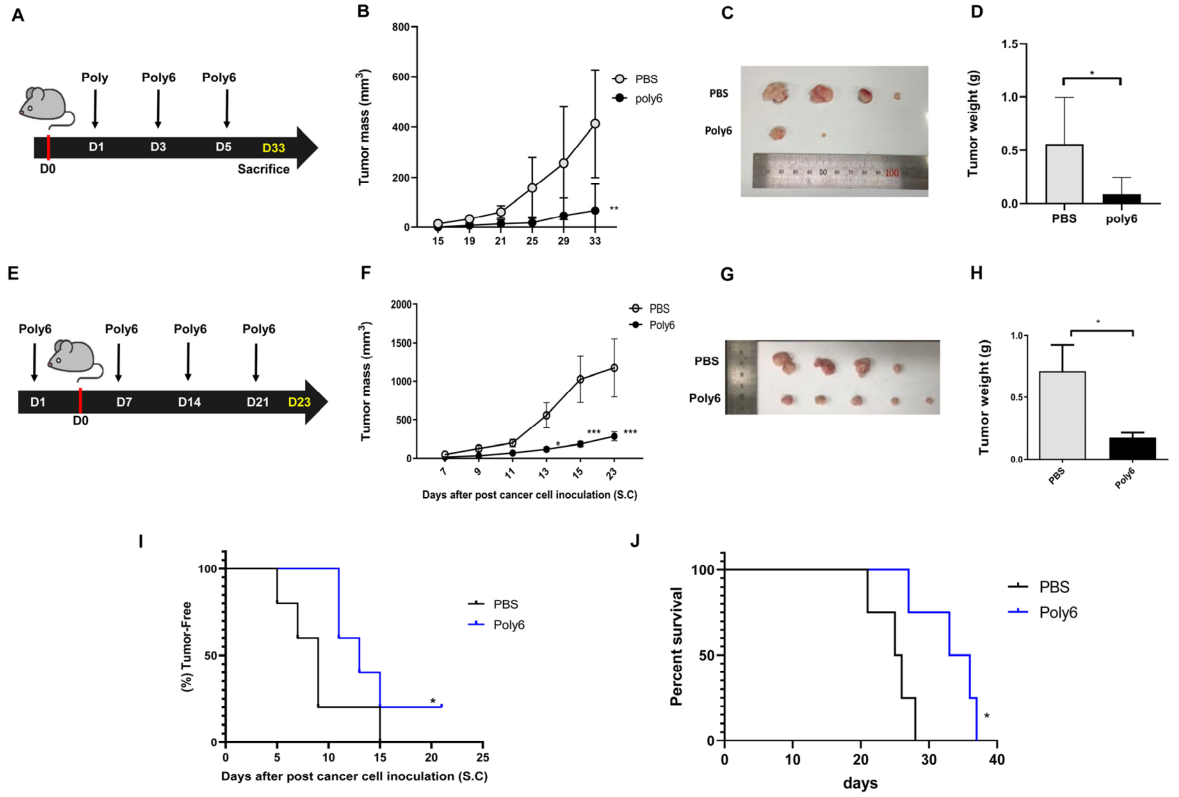

Figure S4. Poly6 exerts anticancer effect on a pancreatic and pre-activation cancer mouse model. (**A**) in vivo schedule of pancreatic cancer model. PanO2 cells were inoculated on day 0, and then Poly6(10 μg) was administrated 3 times (n = 4). (**B**) Comparison of tumor growth between PBS and Poly6 treated group. (**C**) Picture of tumor tissue. Tumor was not found in half of Poly6 treated mouse. (**D**) Weight of tumor tissues. (**E**) Schematic in vivo schedule of pre-activation experiments. Mice were administered Poly6(10 μg) before MC38 cancer cell inoculation. The next day, MC38 cells (1×10^6 cells/100 μL) were subcutaneously inoculated into C57BL/6 mice. After cancer cell injection, Poly6 was administered 3 times more times distant from the site of MC38 injection (PBS; n = 4, Poly6; n = 5). (**F**) Comparison of tumor growth followed by treatment with Poly6. (**G**) Images of tumors extracted from MC38 -bearing mice on day 23. (**H**) Weight of MC38 cancer on day 23. (**I**) Tumor incidence was improved in C57BL/6 mice treated with Poly6 three times after injection of 1×10^6 MC38 cells. The percent of tumor-free mice was indicated (n=5). (**J**) Survival rate was estimated in tumor-bearing mice after Poly6 injection 3 times (n = 4). These results are representative of two independent experiments. Significance differences (* $p < 0.05$, ** $p < 0.01$, *** $p < 0.001$ and **** $p < 0.0001$) among different groups are shown in related figures, and the data are presented as mean ± s.e.m. of mice. Student's t-test, one- and two- way ANOVA were used. Survival test was evaluated by Log-rank test survival analysis.

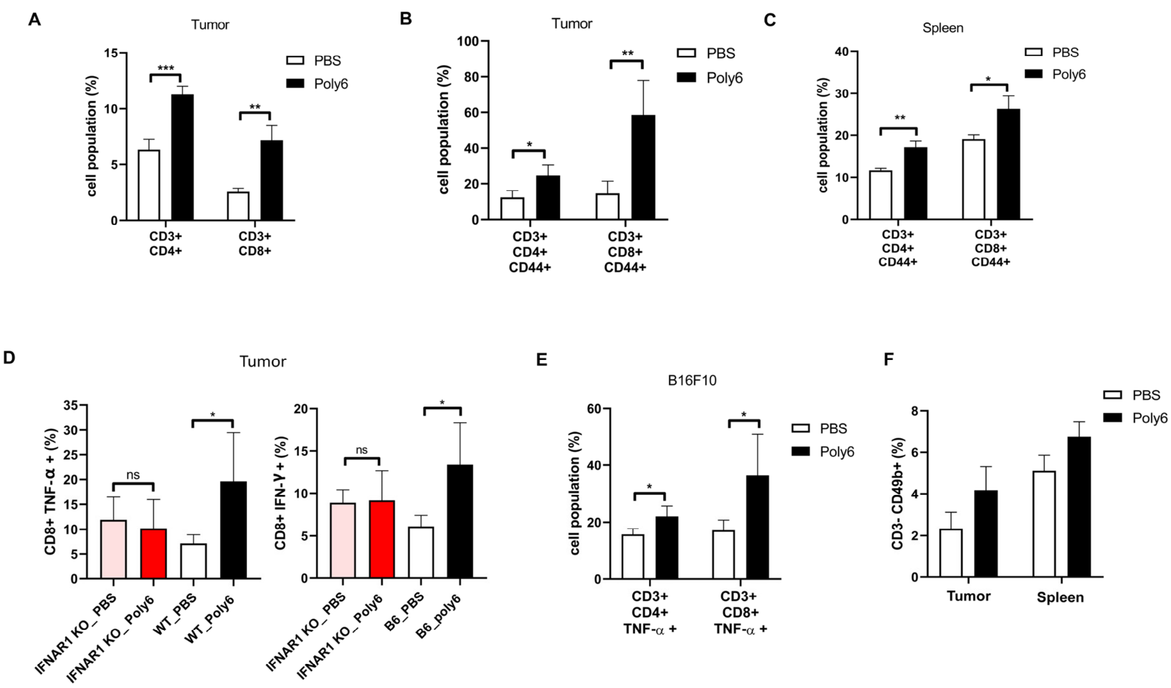

Figure S5. Poly6 exerts anticancer effects via induction of T cell activation in the tumor microenvironment. (**A**) Evaluation of CD4+ and CD8+ T cells populations in MC38 tumor tissue extracted on day 16 ($n = 4$). (**B**) Single cells from dissociated MC38 tumor tissues extracted on day 16 after MC38 injection were stained with anti-CD44 surface antibody, and then CD44+ T cells were analyzed using FACS ($n = 4$). (**C**) The population of activated CD44+ T cells in splenocytes extracted from MC38 tumor-bearing mice on day 16 was assessed by FACS analysis ($n = 4$). (**D**) TNF-α or IFN-γ producing effector CD8+ T cells population in WT and IFNAR1 KO mouse tumor tissues extracted on day 19 ($n = 4$). (**E**) TNF-α producing effector CD4+ and CD8+ T cells in B16F10 tumor tissue extracted on day 12 were analyzed by FACS ($n = 5$). (**F**) The population of CD3− CD49b+ natural killer cells were analyzed in the MC38 tumor tissue extracted on day 16 by FACS ($n = 4$). Significance differences (* $p < 0.05$, ** $p < 0.01$, *** $p < 0.001$ and **** $p < 0.0001$) among different groups are shown in related figures, and the data are presented as mean ± s.e.m. of mice. Student's *t*-test was used.

.

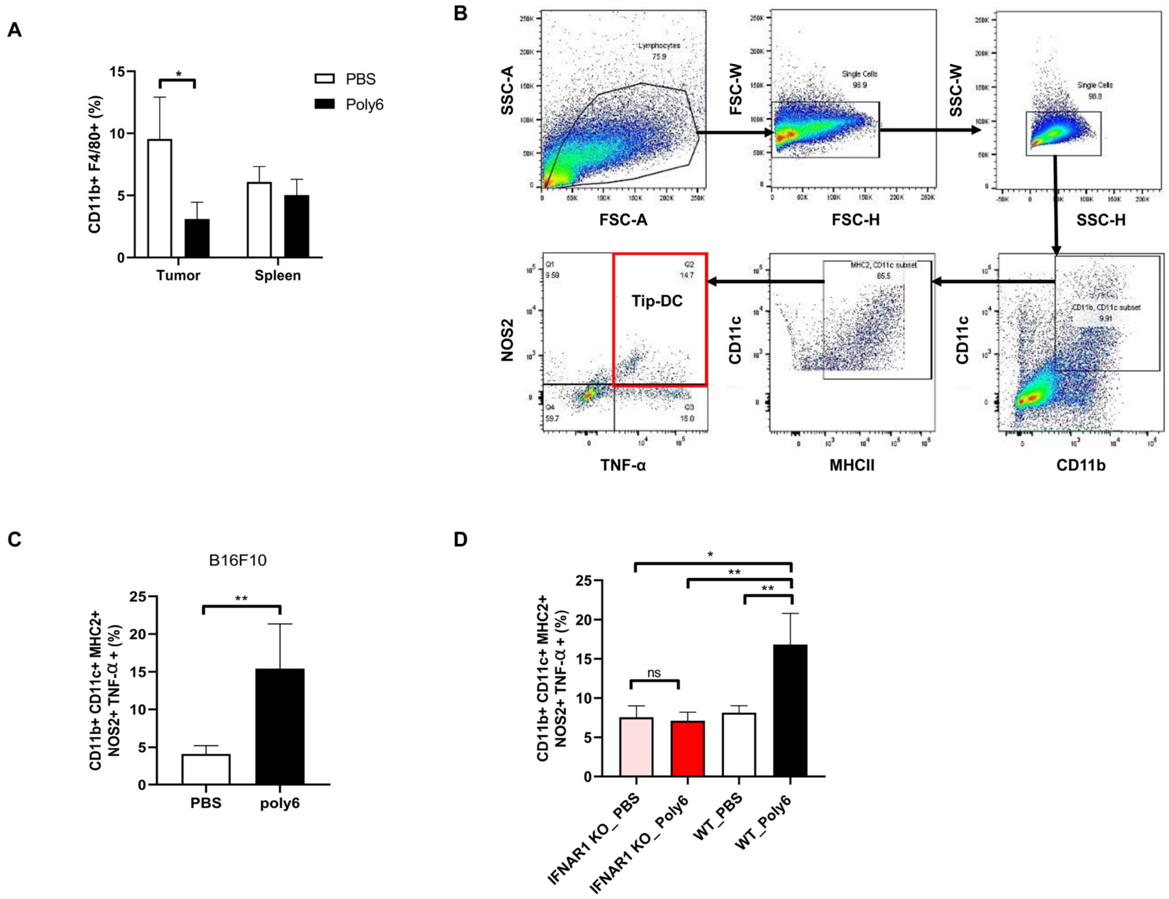

Figure S6. Poly6 induces the generation of Tip-DCs in tumor tissue from a cancer-bearing mouse model. (**A**) Tumor tissue and spleen were extracted on day 16 after MC38 injection. The population of CD11b+ F4/80+ macrophages was analyzed by FACS (n = 4). (**B**) The gating strategy of Tip-DCs was used in tumor tissue, spleen and lymph nodes. This gating was from MC38 tumor tissue on day 16. (**C**) The Tip-DC population in B16F10 melanoma tumor tissues on day 12 was analyzed by FACS (n = 5). (**D**) Tip-DC population in MC38 tumor tissues extracted on day 19 from two mouse models, WT and IFNAR1 KO (n = 4). Significance differences (* $p < 0.05$, ** $p < 0.01$, *** $p < 0.001$ and **** $p < 0.0001$) among different groups are shown in related figures, and the data are presented as mean ± s.e.m. of mice. Student's t-test was used.

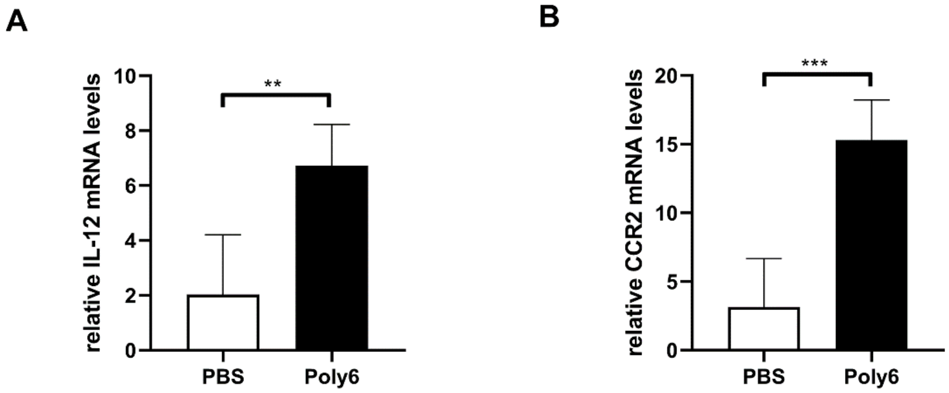

Figure S7. Poly6 treatment can lead to enhanced transcription of IL-12 and CCR2 in tumor Table 12**.** in MC38 tumor tissue was assessed by qRT-PCR. (**B**) The transcription level of CCR2 in MC38 tumor tissue was assessed by qRT-PCR. All primer sequences are indicated in Table 1. Significance differences (* $p < 0.05$, ** $p < 0.01$, *** $p < 0.001$ and **** $p < 0.0001$) among different groups are shown in related figures, and the data are presented as mean ± s.e.m. of mice ($n = 4$). Student's t-test was used.

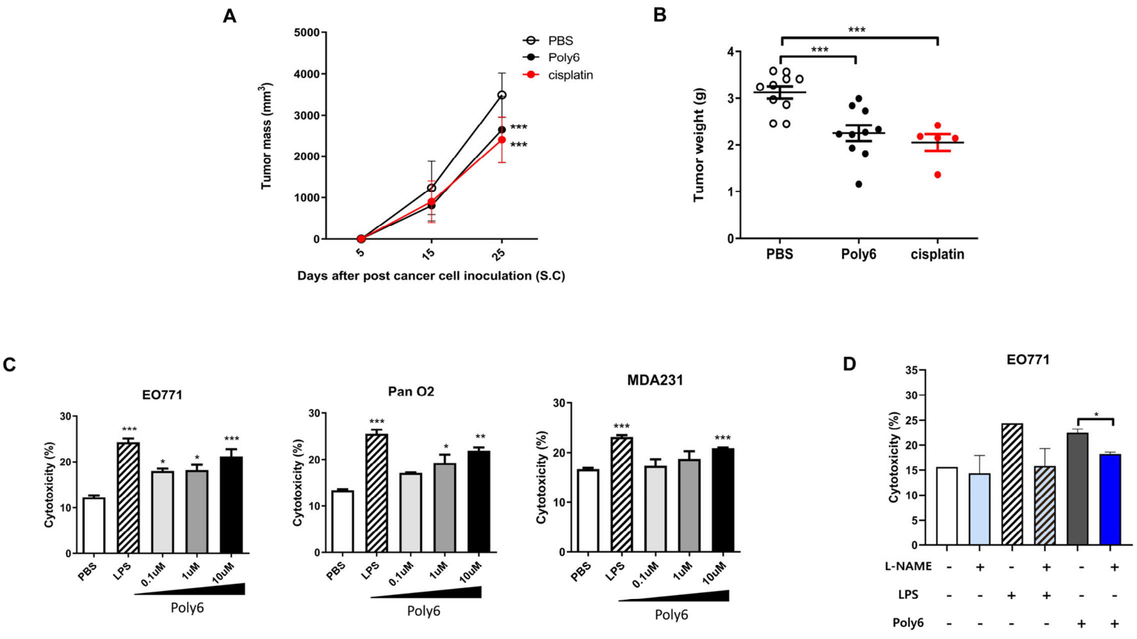

Figure S8. Poly6 induces the direct oncolytic activity of Tip-DCs in a NO-dependent manner. (**A and B**) ObservaTable 4. P-LHB cells (1×10^8) bearing BALB/c nu/nu mice (n = 5~10). (**C**) Cancer cells (PanO2, EO771, MDA231) were analyzed using a coculture system with Tip-DCs that were generated by Poly6. CFSE-labeled cancer cells were cocultured with Poly6 for 48 h in stimulated DC2.4 cells for 4 h. 7AAD+ CFSE-labeled dead cancer cells were analyzed by FACS analysis. (**D**) Inhibited cytotoxicity of EO771 cancer cells by addition of L-NAME was analyzed by FACS. (**C and D**) are representative of two independent experiments; n = 3 biologically independent samples. Significance differences (* $p < 0.05$, ** $p < 0.01$, *** $p < 0.001$ and **** $p < 0.0001$) among different groups are shown in related figures, and the data are presented as mean ± s.e.m.. Student's t-test was used.

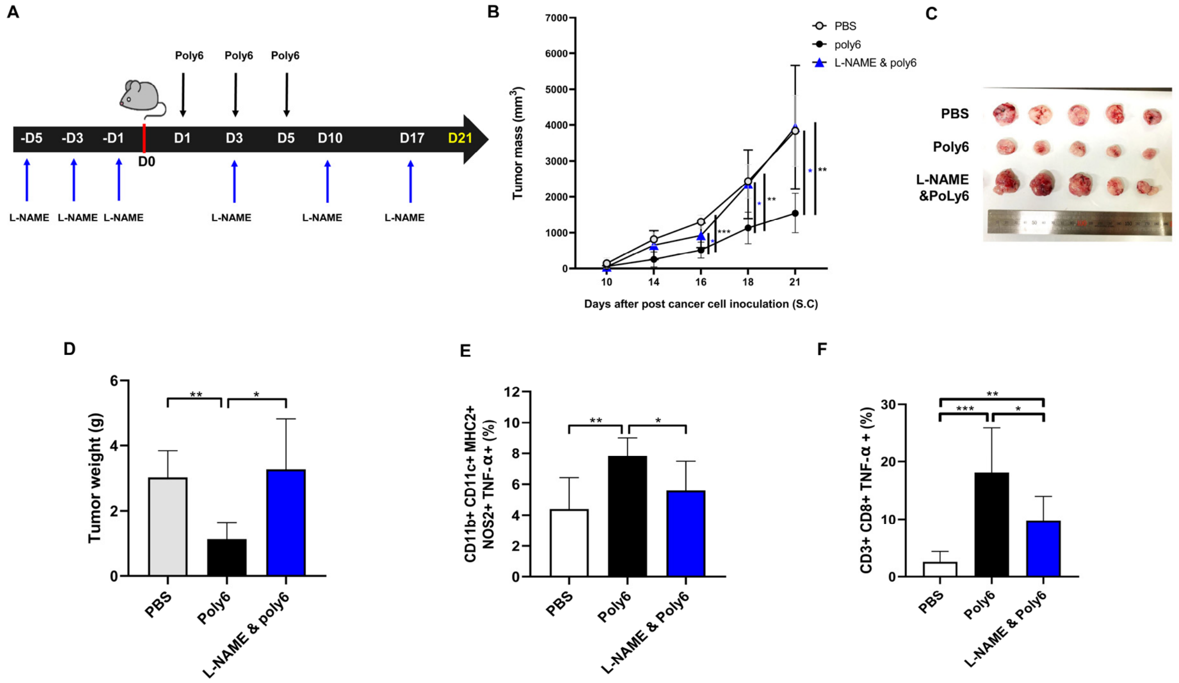

Figure S9. Inhibition of anticancer effect of Poly6 in a tumor-bearing mouse model by L-NAME treatment**.** (**A**) Schematic in vivo schedule for NO dependency of anticancer effect of Poly6. Mice were administered L-NAME (2 mg/100 μL) 3 times via intravenous route before MC38 cancer cell inoculation. After MC38 cells (1×10^6 cells/100 μL) inoculation into C57BL/6 mice, both Poly6 and L-NAME were treated 3 times. (*n*=5). (**B**) Comparison of tumor growth followed by treatment with Poly6 or L-NAME. (**C**) Images of tumors on day 21. (**D**) Weight of MC38 cancer after sacrifice. (**E**) Tip-DC population and (**F**) TNF-*α* producing T cell population of tumor tissues on day 21 analyzed by flow cytometry. Significance differences (* $p < 0.05$, ** $p < 0.01$, *** $p < 0.001$ and **** $p < 0.0001$) among different groups are shown in related figures, and the data are presented as mean ± s.e.m. of mice. Student's *t*-test was used.

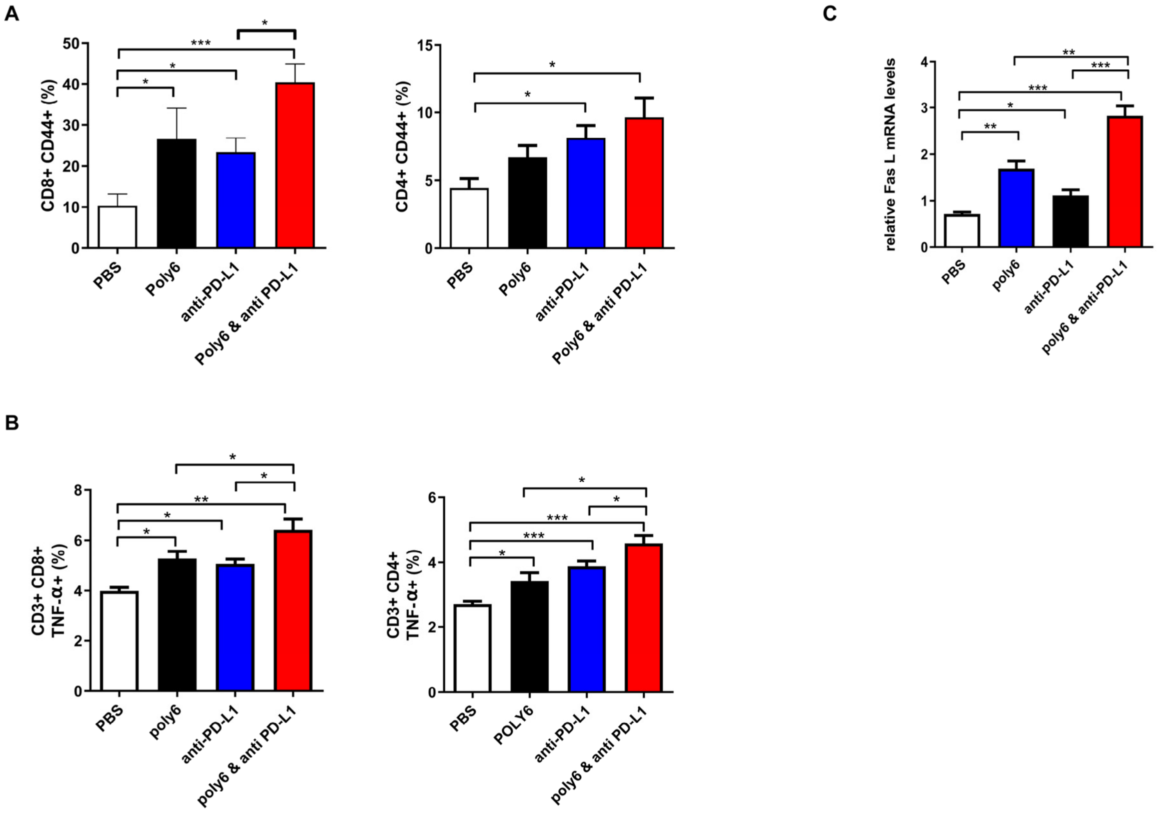

Figure S10. Combination of Poly6 with anti-PD-L1 Ab treatment exerts an enhanced anticancer effect by inducing T cell activation. (**A**) In MC38 tumor tissue on day 21, activated CD44 positive CD4+, CD8+ T cell population was evaluated by FACS analysis. (**B**) The populations of TNF-α or IFN-γ producing CD4+ and CD8+ T cells in the MC38 tumor extracted on day 21 were analyzed by FACS. (**C**) Relative Fas Ligand mRNA levels in MC38 tumor tissue were quantified by qRT-PCR analysis. Significance differences (* $p < 0.05$, ** $p < 0.01$, *** $p < 0.001$ and **** $p < 0.0001$) among different groups are shown in related figures, and the data are presented as mean ± s.e.m. of mice ($n = 4$). Student's t-test was used.

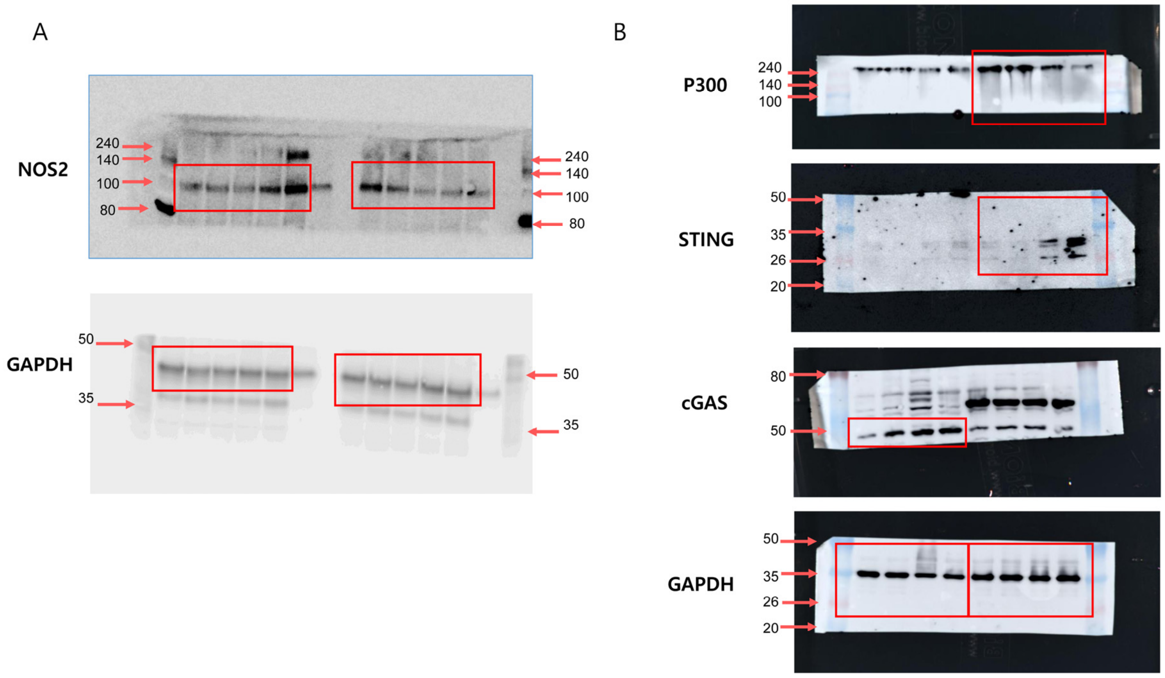

Figure S11 The whole Western blot figures and intensity ratio of each band, normalized by Glyceraldehyde 3-phosphate dehydrogenase (GAPDH)**.** (**A**) Uncropped, full length original blots of cropped image of Figure 1E presented in the manuscript. The blots were performed with the indicated antibodies. The cropped area was indicated with the solid lines. (**B**) Uncropped, full length original blots of cropped image of Supplementary Figure 1A and supplementary Figure.1F presented in supplementary materials. The blots were performed with the indicated antibodies. The cropped area was indicated with the solid red lines.

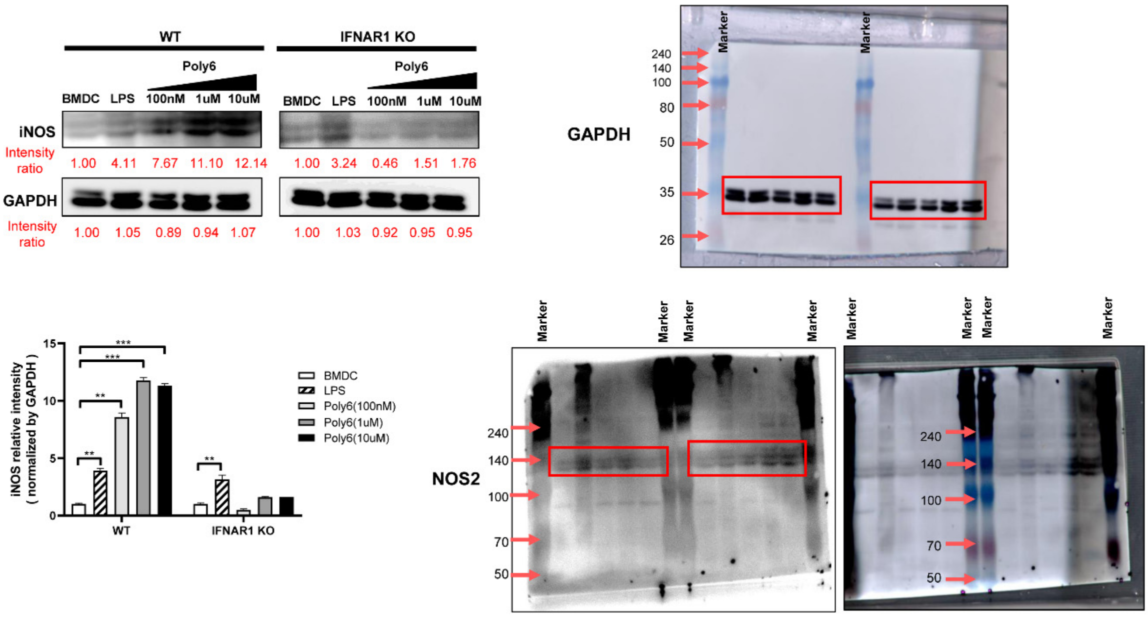

Figure S12. The whole Western blot figures and intensity ratio of each band, normalized by Glyceraldehyde 3-phosphate dehydrogenase (GAPDH). Uncropped, full length additional whole blots of cropped image of Figure 1E presented in the manuscript. The blots were performed with the indicated antibodies. The cropped area was indicated with the solid lines.

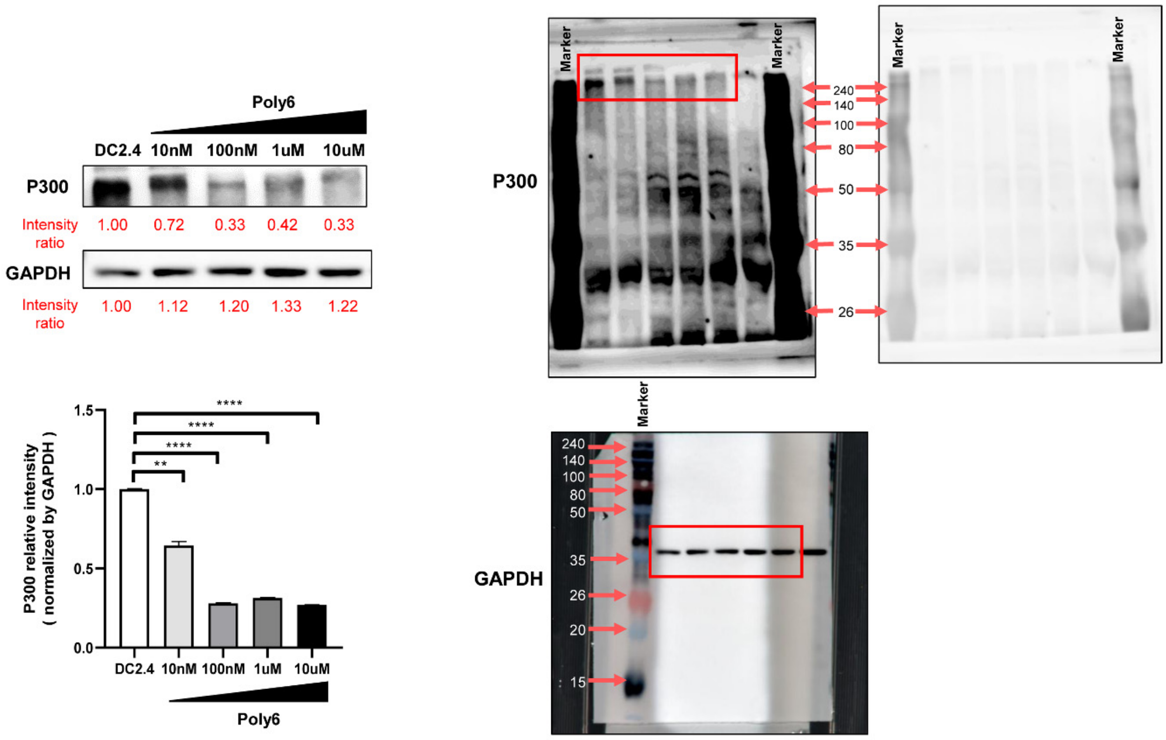

Figure S13. The whole Western blot figures and intensity ratio of each band, normalized by Glyceraldehyde 3-phosphate dehydrogenase (GAPDH). Uncropped, full length additional blots of cropped image of supplementary Figure 1A presented in supplementary materials. The blots were performed with the indicated antibodies. The cropped area was indicated with the solid red lines.

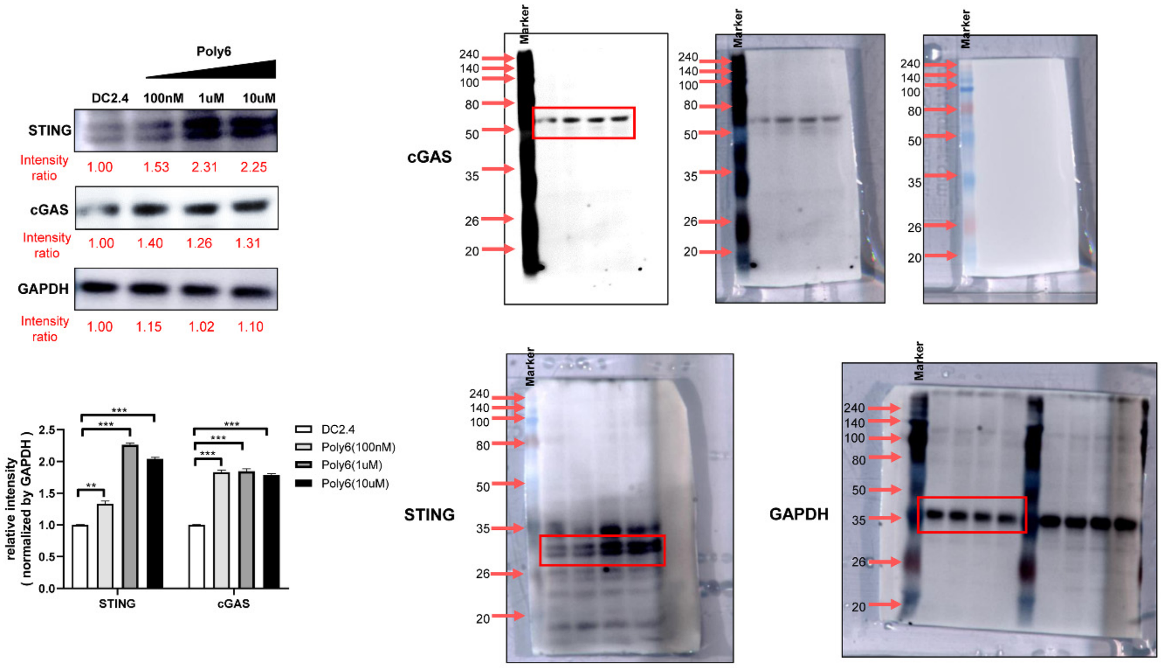

Figure S14. The whole Western blot figures and intensity ratio of each band, normalized by Glyceraldehyde 3-phosphate dehydrogenase (GAPDH). Uncropped, full length additional blots of cropped image of supplementary Figure 1F presented in supplementary materials. The blots were performed with the indicated antibodies. The cropped area was indicated with the solid red lines.

Table S1. Primers used for qPCR.

Genes		Sequence (5′ to 3′)
m FasL	Forward	CGGTGGTATTTTTCATGGTTCTGG
	Reverse	CTTGTGGTTTAGGGGCTGGTTGTT
m TRAIL	Forward	CCTCTCGGAAAGGGCATTC
	Reverse	TCCTGCTCGATGACCAGCT
m Fas	Forward	TCTGGTGCTTGCTGGCTCAC
	Reverse	CCATAGGCGATTTCTGGGAC
m Bak	Forward	TCTGGCCCTACACGTCTACC
	Reverse	ACAAACTGGCCCAACAGA AC
m Bax	Forward	GGAGCAGCTTGGGAGCG
	Reverse	AAAAGGCCCCTGTCTTCATGA
m Bcl2	Forward	ACTTCGCAGAGATGTCCAGTCA
	Reverse	TGGCAAAGCGTCCCCTC
m perforin	Forward	CTGGCTCCCACTCCAAGGTA
	Reverse	GGCTGTAAGHACCGAGATGC
m granzymeB	Forward	CCACTCTCGACCCTACATGG
	Reverse	GGCCCCCAAAGTGACATTTATT
m 18s	Forward	AGTCCCTGCCCTTTGTACACA
	Reverse	CGATCCGAGGGCCTCACTA
mt.Dloop-1	Forward	AATCTACCATCCTCCGTGAAACC
	Reverse	TCAGTTTAGCTACCCCCAAGTTTAA
mt.Dloop-2	Forward	CCCTTCCCCATTTGGTCT
	Reverse	TGGTTTCACGGAGGATGG
mt.ND4	Forward	AACGGATCCACAGCCGTA
	Reverse	AGTCCTCGGGCCATGATT
CCR2	Forward	ATCCACGGCATACTATCAACATC
	Reverse	CAAGGCTCACCATCATCGTAG
IL-12	Forward	CCACTGGAACTACACAAGAACG
	Reverse	GCACAGGGTCATCATCAAAG
